# Sulfonated magnetic spirulina nanobiomaterial as a novel and environmentally friendly catalyst for the synthesis of dihydroquinazolin-4(1H)-ones in aqueous medium

**DOI:** 10.1038/s41598-024-52749-2

**Published:** 2024-01-27

**Authors:** Elahe Mashhadi, Javad Safaei-Ghomi

**Affiliations:** Department of Organic Chemistry, Faculty of Chemistry, University of Kashan, Kashan, Islamic Republic of Iran

**Keywords:** Synthetic chemistry methodology, Heterogeneous catalysis

## Abstract

Spirulina algae is an excellent candidate for catalyst preparation due to its reactive functional groups, cost-effectiveness, widespread commercial accessibility, and biodegradability. In this study, magnetized Spirulina was used for the synthesis of dihydroquinazolin-4(1H)-ones (DHQZs) as catalyst. Magnetized Spirulina was produced by CoFe_2_O_4_ and sulfonation method using chlorosulfonic acid to create the catalyst [CoFe_2_O_4_-Sp-SO_3_H]. It was affirmed by various techniques, including Fourier transform infrared (FT-IR), Vibrating sample magnetometry (VSM), Powder X-ray diffraction (XRD), Energy-dispersive X-ray spectroscopy (EDS), Thermogravimetric analysis (TGA), Transmission electron microscopy (TEM), Field emission scanning electron microscopy (FE-SEM), and elemental mapping techniques. DHQZs synthesis was accomplished through a concise one-pot, three-component reaction involving a range of diverse aldehydes, isatoic anhydride, and primary aromatic amine, within an aqueous medium. The method offers several advantages, including using green conditions, the generation of several new 2-furan-quinazolinone derivatives, chromatography-free purification, short reaction times, appropriate yield of product (75–96%), and catalyst recyclability. The proposed catalyst and water as solvent demonstrated a strong synergistic effect, leading to the prosperous synthesis of various novel dihydroquinazolinones at 60 °C. These numerous benefits make our approach highly attractive for academic research and industrial applications.

## Introduction

In today's era, using renewable and environmentally friendly catalysts has emerged as a cost-effective and clean technology to remove the pollutant^1^. Spirulina platensis, a multicellular, spiral-shaped blue-green alga, has garnered significant attention for its potential applications in the food, cosmetics, and pharmaceutical industries. Its prominence is attributed to its accessibility in the environment, low cost, and the presence of various functional groups^2,3^. The composition of Spirulina is nutrient-rich, encompassing proteins, lipids, carbohydrates, fiber, a range of minerals, vitamins, γ-linolenic acid, carotenoids, chlorophyll, and phycocyanin^4,5^. Thus, functional groups in the Spirulina exhibit chemical reactivity, offering opportunities for introducing additional groups at these sites to undergo structural modifications^6,7^. This, in turn, serves to enhance the catalytic performance of Spirulina. Using toxic, volatile, expensive, and non-recoverable organic solvents contributes significantly to environmental pollution and poses risks to human health. Therefore, there is a growing interest in developing stable catalysts in water that can be easily recycled. Water is a green, cheap, readily available, non-flammable, and non-volatile solvent^8–11^.

**Scheme 1:**
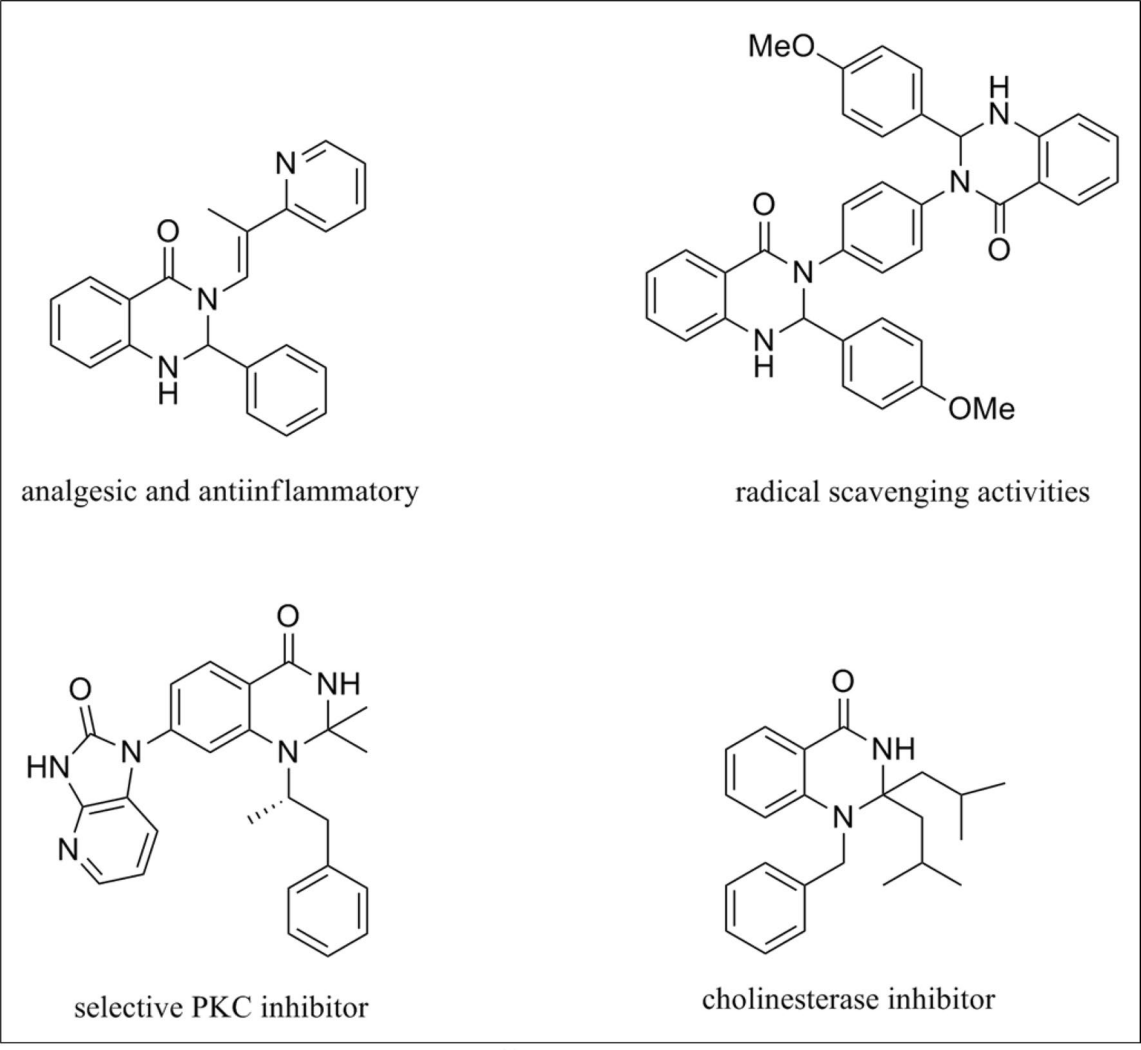
Examples of biological activities of DHQZ derivatives.

Magnetically separable nanocatalysts provide a convenient and efficient solution for isolating catalysts from the reaction mixture. Utilizing an external magnet eliminates the need for complex work-up procedures. Furthermore, these nanocatalysts boast crucial characteristics like high activity, stability, reusability, and environmentally friendly properties, making them significant in green chemistry^12–14^ Dihydroquinazolin-4(1H)-one derivative is a fused heterocyclic compound with various applications in the pharmaceutical industry^15,16^. These compounds exhibit diverse biological activities, anticancer^17^, anticonvulsant^18^ anti-inflammatory^19^, and antimalaria properties^20^. Due to their physiological significance and pharmaceutical potential, developing novel N-heterocyclic molecules is crucial in drug discovery and development. Over the past few decades, the pharmaceutical industry has extensively documented numerous pharmaceutical compounds containing the 2,3-dihydroquinazolin-4(H)-one skeleton (Scheme 1)^21^.

In recent years, there have been numerous reports on the synthesis of 2,3-dihydroquinazolin-4(1H)-ones using diverse catalysts, such as Fe_3_O_4_@SiO_2_@TiO_2_-OSO_3_H^22^, 5,5'-Indigodisulfonic acid^23^, MCM-41-SO_3_H^24^, SCMNPs-Pr-HMTA-SO_3_H^25^, Al(H_2_PO_4_)_3_^26^, CoAl_2_O_4_ Nanoparticles^27^, SnCl_2_.2H_2_O^28^, Fe_3_O_4_@EDTA/CuI^29^, SiO_2_–H_3_PW_12_O_40_^30^, Co aminobenzamid@Al SBA 15^31^, Boric Acid Supported on Montmorillonites^32^. However, most of these procedures have certain limitations, such as lengthy procedures, harsh reaction conditions, hazardous and volatile organic solvents, application of expensive and unavailable reagents, and non-reusability of the catalyst. Driven by the current universal challenges to partake in unpolluted surroundings, our recent research has effectively created a straightforward and environmentally conscious approach for producing 2,3-dihydroquinazolin-4(1H)-ones. This was achieved utilizing a magnetized Spirulina nanocomposite that had been sulfonated, serving as a nanobiocatalyst. Moreover, we have undertaken the synthesis of some novel 2-furan-quinazolinone derivatives.

## Experimental section

### Structural analysis of the sulfonated magnetic Spirulina nanobiomaterial

**Scheme 2:**
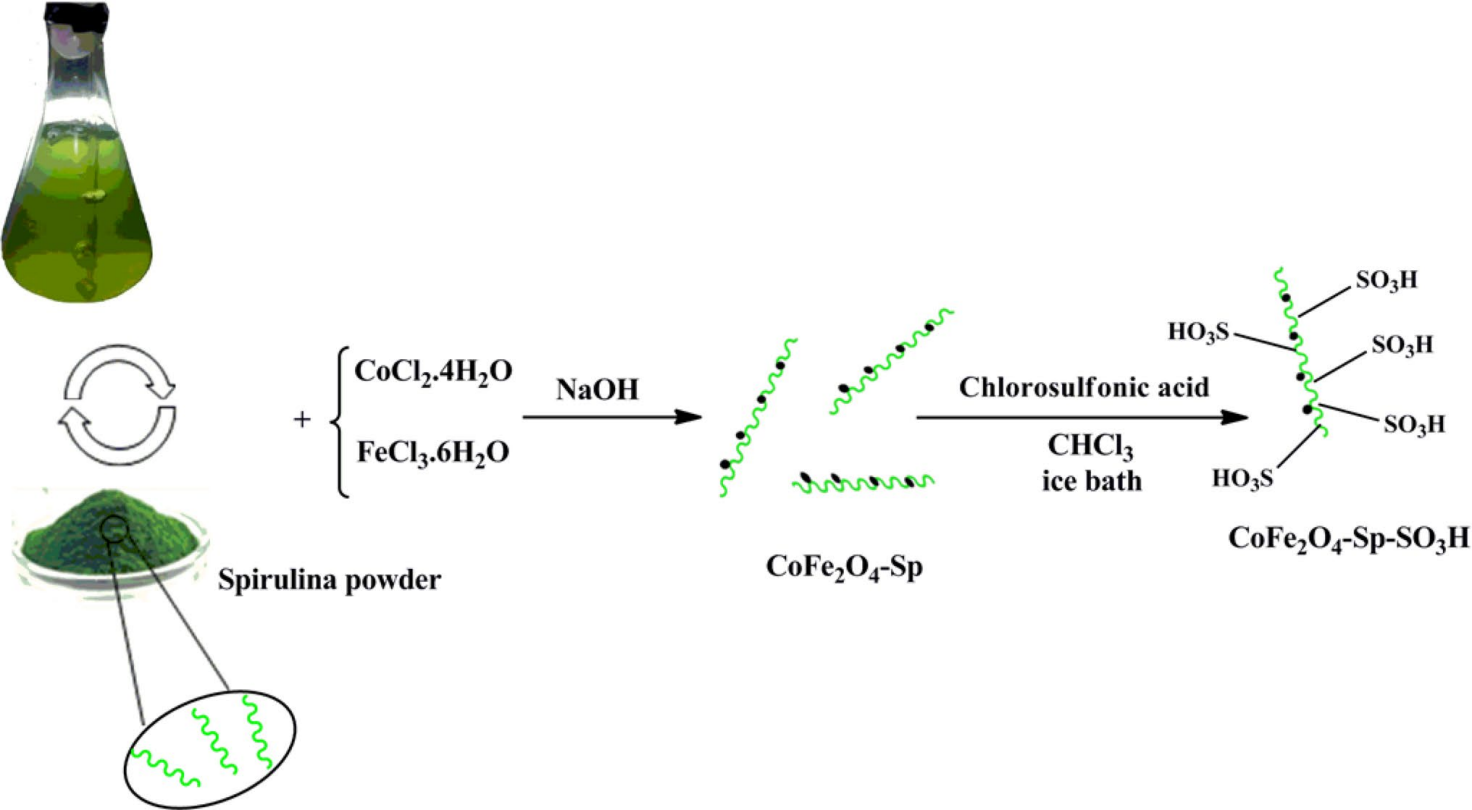
The synthesis of the catalyst CoFe_2_O_4_-Sp-SO_3_H.

In this study, the first step is the preparing of Spirulina Platensis microalgae powder using Zarrouk's culture medium^33^. Fe(III) and Co(II) were dissolved in deionized water (DI). Dry Sp powder was added to the above solution. Subsequently, NaOH was added to the mixture, which was stirred (pH = 11). Finally, chlorosulfonic acid was added to a mixture of CoFe_2_O_4_-Sp in chloroform at 0 °C (Scheme 2).

**Figure 1:**
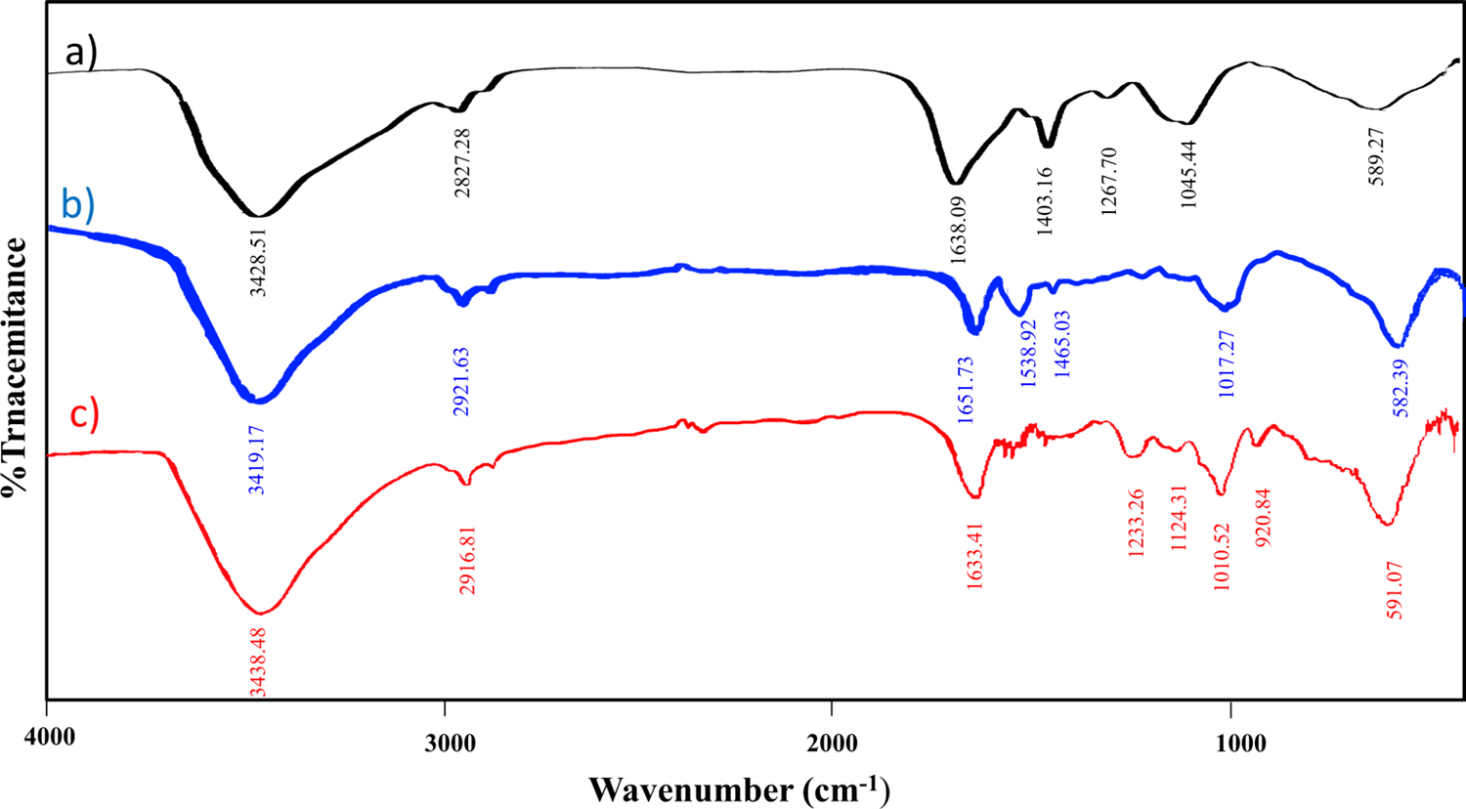
IR spectra of Spirulina (**a**) CoFe_2_O_4_-Sp (**b**) CoFe_2_O_4_-Sp-SO_3_H (**c**).

IR analysis was carried out to determine and describe the functional groups shown in Fig. 1. In the FT-IR spectrum of Spirulina (depicted in Fig. 1b), the central peak at 3428 cm^−1^ corresponds to hydroxyl (-OH) and amino (-NH) groups. Peaks in the 1645–1570 cm^−1^ range indicate the N–H bending vibration of secondary amide. The bending vibration of CH_2_ can be seen in peaks ranging from 1430 to 1400 cm^−1^. Moreover, specific frequencies spanning 1300–1240 cm^−1^ show the C–O stretching of alcohol and O–H bending. In the same frequency range 1300–1250 cm^−1^, there is the presence of carbonyl asymmetric C–O–C ester stretching, along with another peak range of 1115–1025 cm^−1^ indicating symmetric C–H stretching^34^. The FT-IR analysis of CoFe_2_O_4_-Sp confirms the successful synthesis of the magnetic nanocomposite. A peak at 582 cm^−1^ confirms the presence of the iron–oxygen bond. Furthermore, in the analysis of CoFe_2_O_4_-Sp-SO_3_H, the peak at 1233 cm^−1^ corresponds to the sulfur-oxygen (S=O) bond.

**Figure 2:**
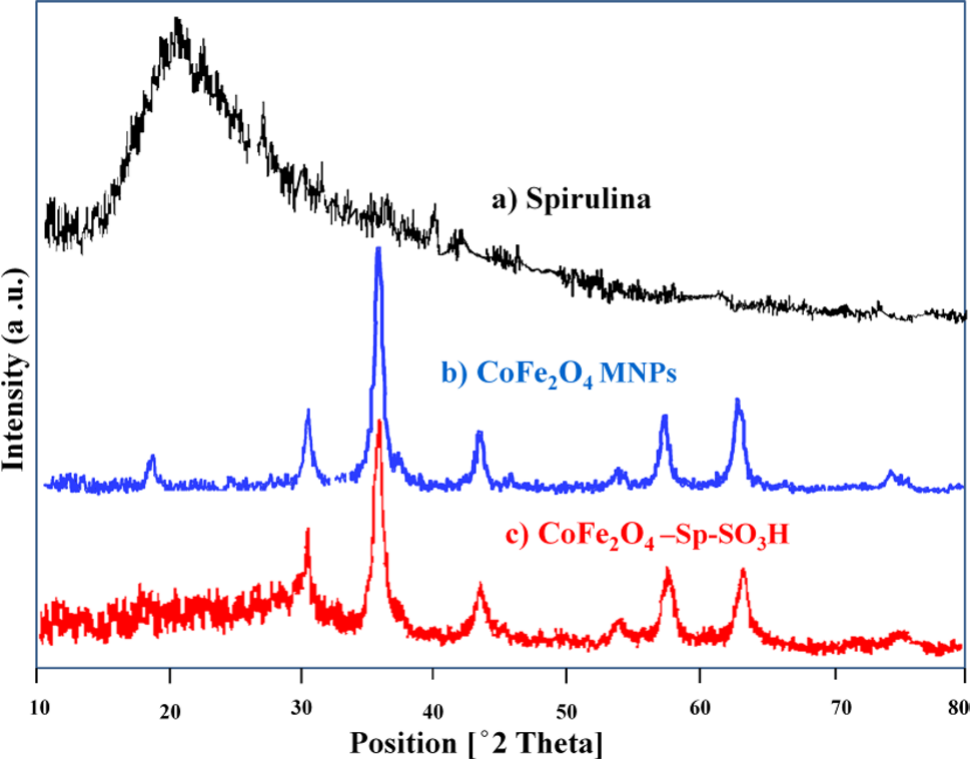
XRD patterns for Spirulina (**a**), CoFe_2_O_4_ MNPs (**b**), CoFe_2_O_4_-Sp-SO_3_H (**c**).

The X-ray diffraction (XRD) analysis was performed to examine the structures of CoFe_2_O_4_ MNPs, Spirulina algae, and CoFe_2_O_4_-Sp-SO_3_H. Figure 2 shows the XRD pattern. The characteristic peaks of CoFe_2_O_4_ were detected at 2θ angles of 74.5°, 63.0° 57.3°, 53.9°, 43.3°, 35.8°, 30.4°, and 18.3°, which correspond to the (533), (440), (511), (422), (400), (311), (220), and (111) crystal planes of CoFe_2_O_4_. These peaks closely matched the standard spectra^35^, confirming the presence of CoFe_2_O_4_ crystal in the samples. The XRD pattern of CoFe_2_O_4_-Sp-SO_3_H exhibited peaks at similar positions as those of CoFe_2_O_4_, confirming the fact of CoFe_2_O_4_ crystalline structure in the final product. Additionally, weak broad bands between 15° and 30° indicated the presence of amorphous sulfonated Spirulina in the product.

**Figure 3:**
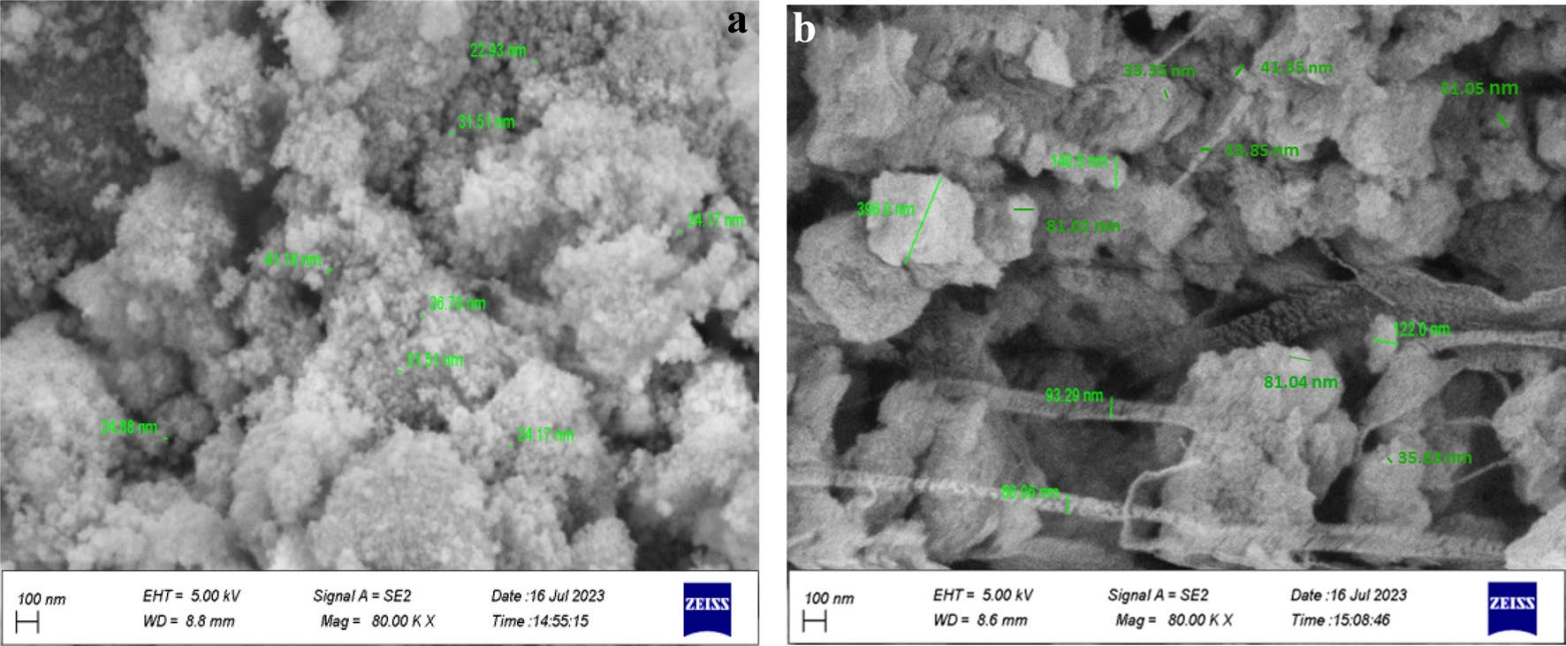
FE-SEM images of CoFe_2_O_4_ (**a**), CoFe_2_O_4_-Sp-SO_3_H (**b**).

The morphology and distribution of particle sizes in the synthesized CoFe_2_O_4_ and CoFe_2_O_4_-Sp-SO_3_H nanocomposite were studied using FE-SEM analysis (refer to Fig. 3). It is evident from the images that both CoFe_2_O_4_ and CoFe_2_O_4_-Sp-SO_3_H possess nano-sized structures, with average sizes of approximately 28 nm and 97 nm, respectively. The findings distinctly demonstrate that the Spirulina microalgae served as a biotemplate and has been effectively coated by magnetic nanoparticles, displaying slight agglomeration (Fig. 3b).

**Figure 4:**
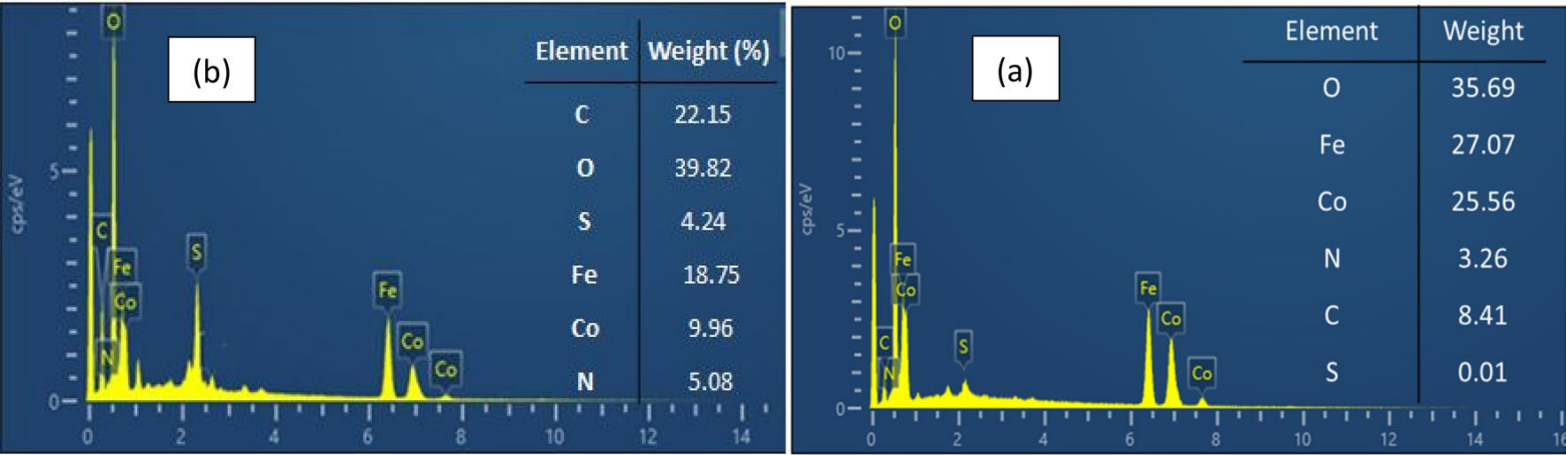
The EDX spectra of CoFe_2_O-Sp (**a**), CoFe_2_O_4_-Sp-SO_3_H (**b**).

The elemental compositions are determined by analyzing the energy-dispersive X-ray (EDX) spectrum. As shown in Fig. 4a, the fundamental makeup of CoFe_2_O_4_-Sp MNPs comprises Cobalt (Co), Oxygen (O), Carbon (C), Iron (Fe), Nitrogen (N) and sulfure (S). Since the composition of spirulina algae includes C, H, N, S and O^33^, then the presence of Nitrogen (N), Sulfure (S) and Carbon (C) suggests that a coating of Spirulina algae was applied to the surface of the CoFe_2_O_4_ nanoparticles. It's crucial to emphasize that the highest proportion of atoms is linked to oxygen. This element is found in Spirulina microalgae as an organic component and is also present in the magnetic nanoparticles as an inorganic element of the synthetic catalyst.

As illustrated in Fig. 4b, roughly 4.2% of the total weight of the CoFe_2_O_4_-Sp-SO_3_H nanocomposite is accounted for by sulfur. This provides further confirmation of the successful execution of the sulfonation process.

**Figure 5:**
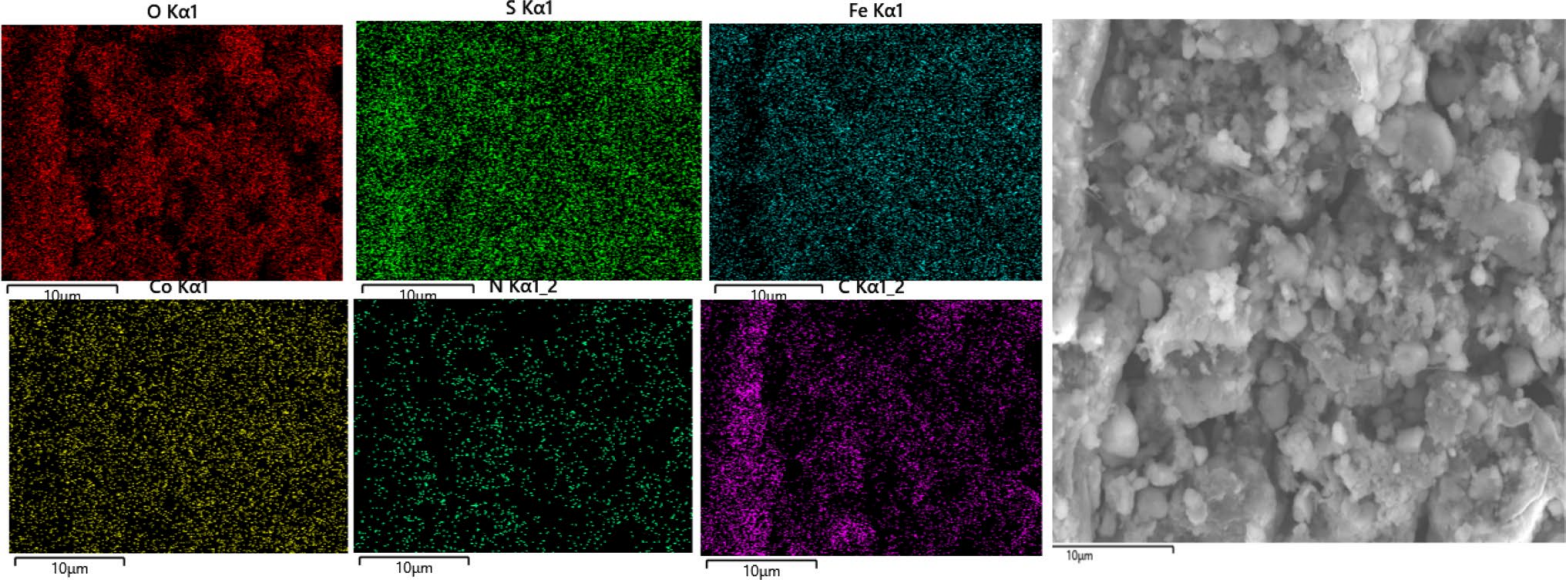
The elemental mapping of CoFe_2_O_4_-Sp-SO_3_H.

The elemental mapping images depict a homogeneous distribution of all elements within the CoFe_2_O_4_-Sp-SO_3_H structure (Fig. 5).

**Figure 6:**
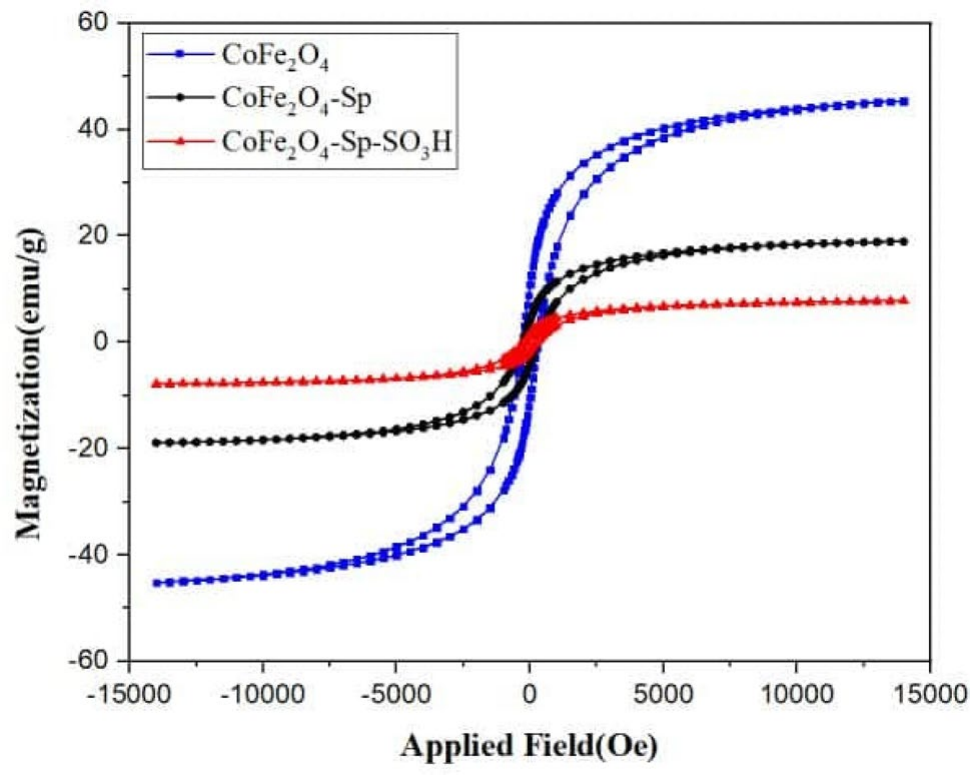
The VSM curve for different synthesis parts of the catalyst.

The VSM curve for the catalyst is given in Fig. 6. The levels of saturation magnetization for CoFe_2_O_4_, CoFe_2_O_4_-Sp, and CoFe_2_O_4_-Sp-SO_3_H are 44.05 emu g^−1^, 18.49 emu g^−1^, and 10.50 emu g^−1^, respectively.The significant decline in saturation magnetization within the CoFe_2_O_4_-Sp nanocomposite results from the surface coating applied to the CoFe_2_O_4_ nanoparticles. As depicted, the magnetic characteristics of the CoFe_2_O_4_-Sp experienced a minor reduction (approximately 8.0 emu g^−1^) after the sulfonation process. The most likely explanation is the removal of specific CoFe_2_O_4_ magnetic nanoparticles that were not firmly bonded to the Spirulina during the sulfonation procedure. Notwithstanding this reduction in the catalyst's saturation magnetization, this value remains sufficient to facilitate a practical magnetic separation process.

**Figure 7:**
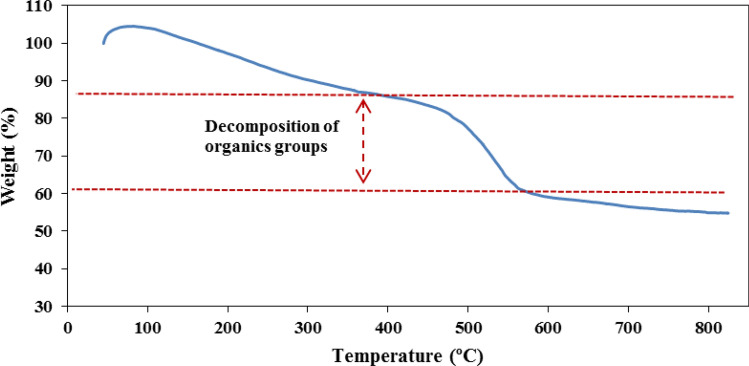
The TGA curves of CoFe_2_O_4_-Sp-SO_3_H.

The TGA for the sulfonated magnetic Spirulina nanobiomaterial (CoFe_2_O_4_-Sp-SO_3_H) are displayed in Fig. 7. In the initial stage, there is a rise in weight percentage, attributed to the buoyancy effect within the TGA apparatus^36^. Within the broad temperature range of 460–580 °C, a notable 25% mass loss is observed, primarily ascribed to the decomposition of organic groups, providing further evidence of the presence of algae. The thermal analysis results indicate that the nanocatalyst exhibits impressive thermal stability, nearly reaching temperatures of up to 600 °C.

A series of experiments were conducted to find the best conditions for the reaction. These experiments involved altering factors like the solvent used, the quantity of catalyst, and the temperature. The standard reaction involved isatoic anhydride, 4-methylaniline, and 4-chlorobenzaldehyde. Initially, the impact of different solvents (EtOH, CH_3_CN, DMF, MeOH, H_2_O, and DCM) on the model reaction was investigated. It was observed that water yielded an excellent product with high efficiency in a short period (see Table 1, Entries 8–13).

**Table 1:** Optimized conditions to prepare dihydroquinazolin‑4(1H)‑ones.

Entry	Solvent	Catalyst (g)	Time (min)	Temperature (°C)	Yield^a^ (%)
**1**	H_2_O	–	30	60	25
**2**	H_2_O	0.05	60	60	92
**3**	H_2_O	0.05	30	80	92
**4**	H_2_O	0.05	30	40	80
**5**	H_2_O	0.05	30	25	72
**6**	H_2_O	0.02	30	60	85
**7**	H_2_O	0.08	30	60	91
**8**	H_2_O	0.05	30	60	91
**9**	MeOH	0.05	30	60	76
**10**	EtOH	0.05	30	60	80
**11**	DMF	0.05	30	60	20
**12**	DCM	0.05	30	60	20
**13**	CH_3_CN	0.05	30	60	60

^a^Isolated yields.

On the other hand, when the reaction was carried out without a catalyst, only a 25% yield of the desired product was obtained (Table 1, Entry 1). Referring to the data presented in Table 1, the quantity of the catalyst was fine-tuned, and it was observed that 0.05 g of the catalyst was adequate to achieve a complete yield of 2,3-dihydroquinazolin-4(1H) one (Table 1, Entry 8 compared to Entries 7 and 6). Despite prolonging the reaction time, the yield was no significant increase (Table 1, Entry 2).

Furthermore, the impact of temperature on the model reaction was investigated (Entries 3–5), revealing that a favorable yield could be obtained at 60 °C.

After identifying the optimal reaction conditions, the scope of this reaction was expanded by utilizing different aromatic aldehydes and amines to demonstrate the general applicability of the reaction conditions (Table 2, **2a**–**2l**).

**Table 2:** Synthesis of dihydroquinazolin‑4(1H)‑ones.


Entry	Product	Yield (%)	M.P. /M.P. (^o^C)^b^
**1**		93	205–206/207–209^27^
**2**		95	213–215/216–218^27^
**3**		94	223–224/221–223^27^
**4**		92	224–226/223–225^27^
**5**		92	204–206/205–207^27^
**6**		96	215–217/211–213^37^
**7**		91	273–275/273–275^38^
**8**		96	250–252/247–250^39^
**9**		96	209–211/205–207^40^
**10**		87	187–189
**11**		89	182–184
**12**		85	175–177

^a^Isolated yields.

^b^Literature references.

As anticipated, the intended products were obtained in satisfactory to excellent yields. Additionally, 5-Aryl-2-furaldehydes were studied with different anilines, resulting in superior yields of products within a slightly longer timeframe (Table 3, **3a**–**3f**).

**Table 3:** Synthesis of new 2-furan-quinazolinone derivatives plausible mechanism.


Entry	Product	Yield (%)	M.P. (^o^C)
**1**		77	181–183
**2**		76	162–163
**3**		82	161–164
**4**		75	182–184
**5**		81	160–162
**6**		80	128–130

^a^Isolated yields.

**Scheme 3:**
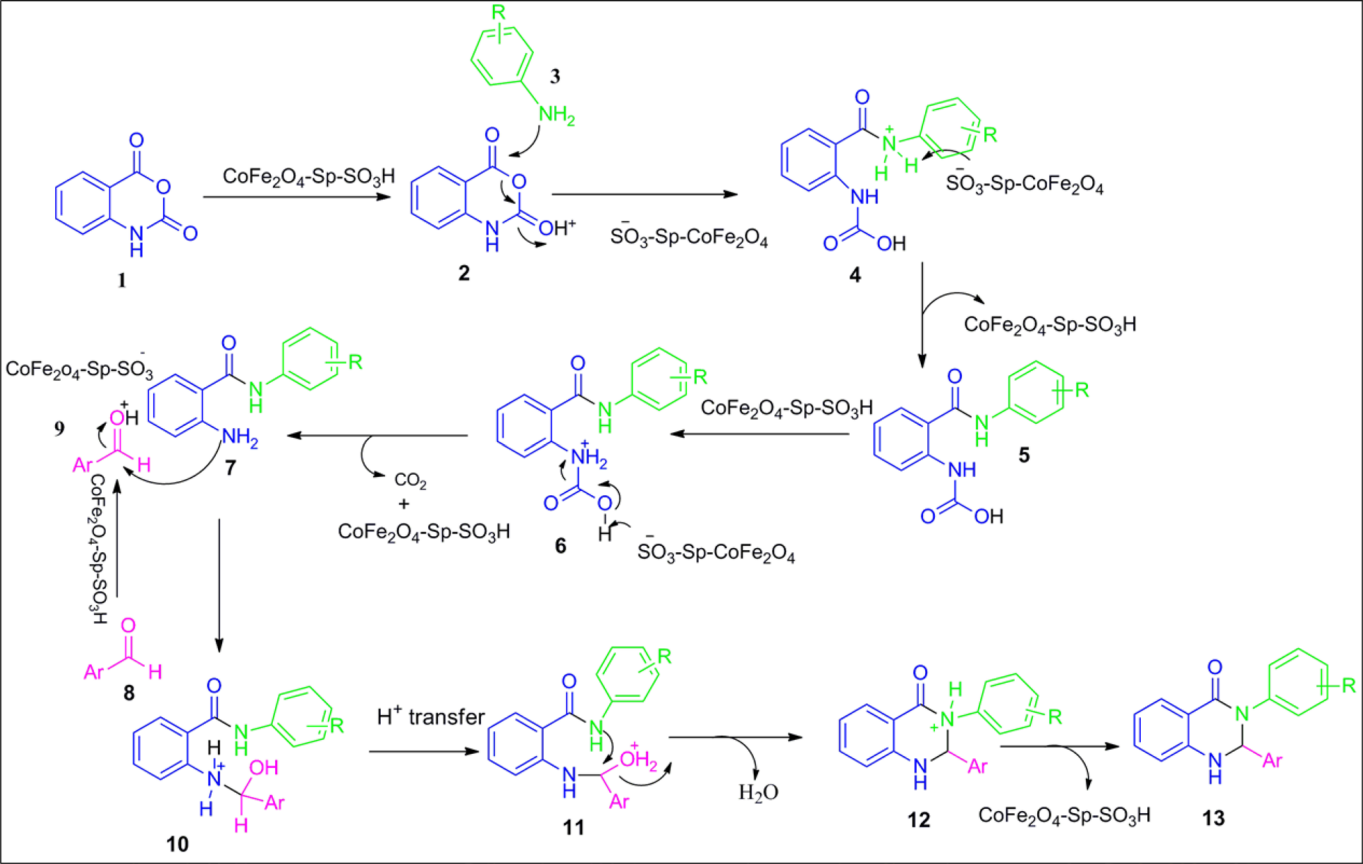
The rational mechanism for dihydroquinazolin-4(1H)-ones.

According to experimental observations and also other mechanisms reported in the literature^11^, a well-founded mechanism and catalytic cycle for synthesizing Dihydroquinazolin-4(1H)-ones using CoFe_2_O4-Sp-SO_3_H are illustrated in Scheme 3. To initiate the process, CoFe_2_O_4_-Sp-SO_3_H activates Isatoic anhydride 1, forming an intermediate denoted as 2. Subsequently, the carbonyl moiety of intermediate 2 undergoes an attack by N-nucleophilic amine 3, yielding intermediate 4. This, in turn, progresses to form intermediate 5. Meanwhile, in the presence of CoFe_2_O_4_-Sp-SO_3_H, the reaction produces intermediate 6, which, through decarboxylation, transforms into substituted-2-aminobenzamide 7. Concurrently, CoFe_2_O_4_-Sp-SO_3_H triggers the activation of aldehyde 8, generating intermediate 9. Following this, the interaction between intermediate 9 and 7 ensues, creating intermediate 10. A proton transfer within intermediate 10 prompts the generation of intermediate 11. Finally, a ring closure via dehydration concludes in the production of intermediate 12, yielding the desired end product compound 13.

### Reusability of the catalyst

**Figure 8:**
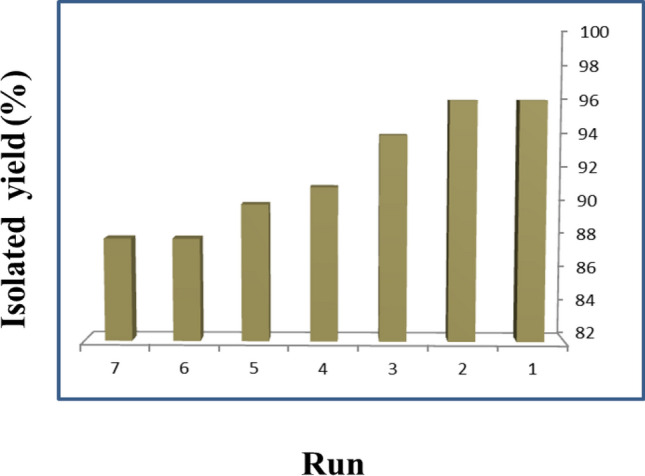
Recycling values for CoFe_2_O_4_-Sp-SO_3_H.

The capacity to reclaim and reuse heterogeneous catalysts is a crucial aspect, carrying substantial significance from both industrial and environmental standpoints. Upon the completion of the reaction, the catalyst was separated utilizing an external magnet and subsequently cleansed with acetone to eliminate the reaction byproducts. These procedures were conducted to assess the recyclability and practical properties of the obtained nanobiocatalyst. The findings depicted in Fig. 8 reveal that the product yield remains relatively stable even after six consecutive runs.

**Figure 9:**
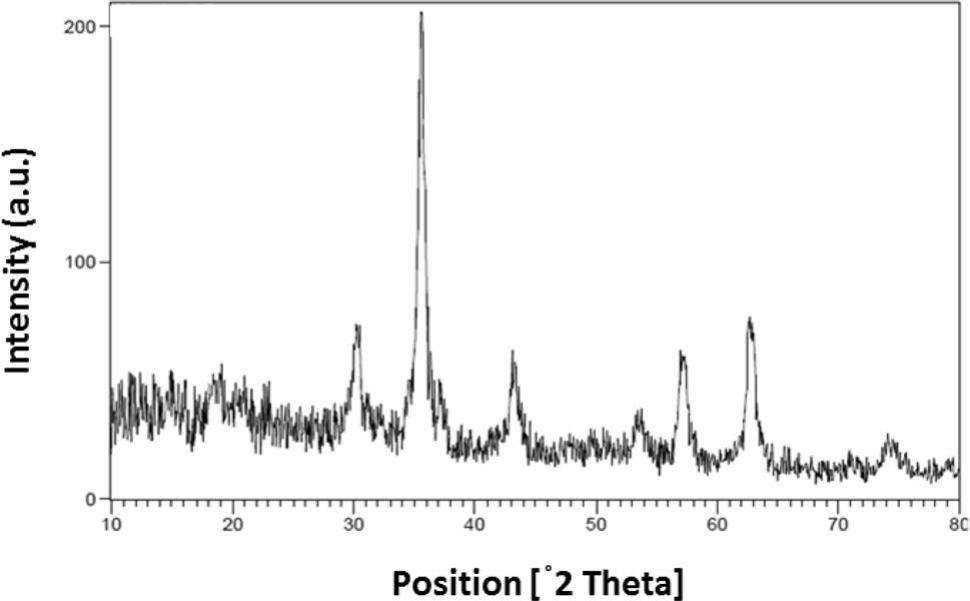
XRD of recycled after seven times CoFe_2_O_4_-Sp-SO_3_H.

**Figure 10:**
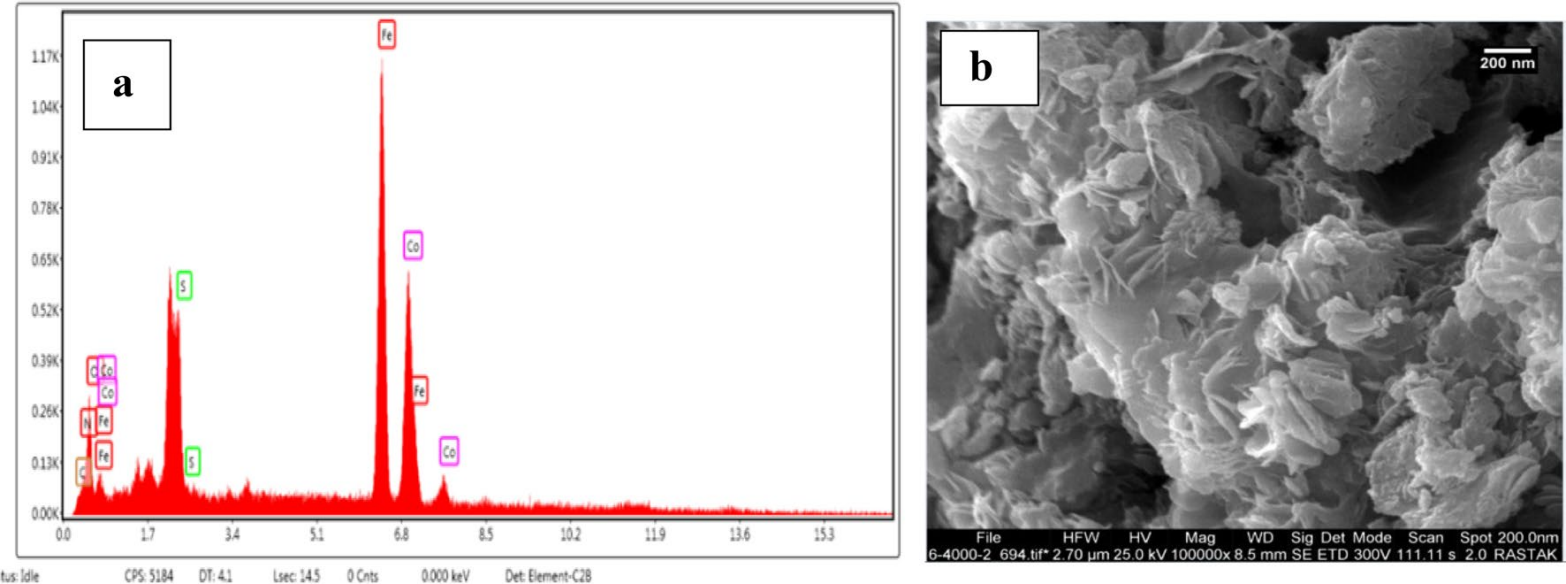
EDX (**a**) and SEM (**b**) of recycled after seven times CoFe_2_O_4_-Sp-SO_3_H.

The nature of the recovered catalyst was investigated by XRD (Fig. 9), EDX and SEM (Fig. 10) analysis. It was observed that the catalyst can be recycled without any significant changes in its structure.

The findings from this investigation and similar research on the model reaction indicate that our method, employing a CoFe_2_O_4_-Sp-SO_3_H catalyst, achieves a greater yield in a shorter times (Table 4).

**Table 4:** Comparison the various catalysts for the synthesis of dihydroquinazolin‑4(1H)‑ones.

Entry	Catalyst	Solvent	Time (min)	Yield (%)	References
**1**	CoFe_2_O_4_	H_2_O	60	75	This work
**2**	CoFe_2_O_4_-Sp	H_2_O	60	75	This work
**3**	Spirulina	H_2_O	60	Trace	This work
**4**	CoFe_2_O_4_-Sp-SO_3_H	H_2_O	30	91	This work
**5**	CoAl_2_O_4_ Nanoparticles	EtOH	120	89	^27^
**6**	Fe_3_O_4_	H_2_O	300	73	^39^
**7**	KAl(SO_4_)_2_.12H_2_O	H_2_O	240	79	^41^

### Substances and methods

In this experiment we used top-quality reagents and reactants sourced from reputable commercial suppliers, ensuring excellent purity. The IR spectra of the produced compounds and various compound which were prepared in the catalyst synthesis process were observed using an FT-IR Magna spectrometer 550 Nicolet with KBr plates. For the ^13^C and ^1^H NMR spectra, we employed a Bruker Avance-400 MHz spectrometer in DMSO or CDCl_3_, utilizing tetramethylsilane as internal reference. To determine the melting points of the products, we utilized the precise Electrothermal 9200 apparatus. For the magnetic measurements of CoFe_2_O_4_-Sp and CoFe_2_O_4_-Sp-SO_3_H, we conducted a thorough analysis using a magnetometer (VSM, PPMS-9T) at 300 K, performing these measurements at the Kashan University in Iran.

We carefully analyzed Powder X-ray diffraction (XRD) using a Philips diffractometer from X'pert Company, utilizing monochromatized Cu Kα radiation (wavelength = 1.5406 Å). We utilized cutting-edge FE-SEM imaging and EDX analysis to visualize the detailed microstructural features, expertly performed using the SIGMA VP-500 (ZEISS) Oxford Instruments Field Emission Scanning Electron Microscope. For thermogravimetric analysis (TGA) data, our Bahr STA-503 instrument facilitated precise measurements under a controlled N_2_ atmosphere, employing a heating rate of 10 °C min^−1^ to ensure accurate results. To assess the purity of substrates and monitor reactions effectively, we relied on the tried-and-tested thin-layer chromatography (TLC) technique, employing silica gel SILG/UV 254 plates from Merck & Co. Elemental analysis was performed on a LECO CHN 923 analyzer. The scanning electron micrograph for recycled catalyst was obtained by SEM instrumentation (SEM, XL-30 FEG SEM, Philips, at 20 kV).

### Spirulina platensis microalgae powder preparation

Spirulina platensis was cultivated using Zarrouk's culture medium, which contains all necessary nutrients. The batch cultivation process involved placing the strain in 1-L glass bottles, including 1 L of the Zarrouk culture medium. The cultivation was carried out under constant light intensity, and the growth medium's pH was maintained around 8 to 11. This cultivation's most suitable temperature range was between 30 and 35 °C. The final biomass was harvested when it reached the desired cell density, typically taking around 12–14 days. The inoculum was filtered to collect the biomass. Then, the resulting product was placed in an oven overnight at 40 °C to obtain the dry biomass of Spirulina.

### Fabrication of CoFe_2_O_4_-Sp

To initiate the procedure, 10 mmol of FeCl_3_.6H_2_O and 5 mmol of CoCl_2_.4H_2_O were dissolved in 50 mL of deionized water (DI). After that, 0.25 g of Sp powder that had been dried was dispersed in 10 mL of DI water using ultrasonication and then added to the solution mentioned above. The pH was subsequently raised to around 12 using a solution of 0.3 M NaOH. The mixture was stirred at a temperature of 80 °C for an hour. After cooling the mixture to room temperature, the resulting residue was separated using an external magnet. The separated solid was then subjected to multiple washes with deionized water until the solution reached a neutral pH level. Lastly, the solid residue was washed with ethanol and dried in an oven at 60 °C.

### Fabrication of CoFe_2_O_4_-Sp-SO_3_H

In a 50 mL round bottom flask, CoFe_2_O_4_-Sp particles (0.5 g) were dispersed in chloroform (10 mL), and the temperature was lowered using an ice bath. Separately, chlorosulfonic acid (99%) (4 mmol) was mixed with chloroform (2.0 mL), and the resulting solution was carefully added drop by drop into the main reaction flask while stirring. Once the addition had been completed, the ice bath was taken away, and stirring was vigorously sustained for 2 h at room temperature. Subsequently, the resulting residue was isolated using a magnet, washed with dichloromethane, and dried to produce Sulfonated Magnetic Spirulina as a brown powder.

### The typical procedure for the synthesis of 2,3-dihydroquinazolin-4(1*H*)-ones

To begin the reaction, a mixture of aromatic amine (1.2 mmol), isatoic anhydride (1 mmol), and CoFe_2_O_4_-Sp-SO_3_H (0.05 g) as a catalyst in water (5 mL) were added, respectively. After allowing the reaction to proceed for 5 min, a diverse aldehyde (1 mmol) was attentively added to the mixture.

### Spectral and analytical data of new compounds

#### 3-(4-bromophenyl)-2-(m-tolyl)-2,3-dihydroquinazolin-4(1H)-one (2j)

White solid; IR (KBr) ν (cm^−1^): 3301 (N–H), 1632 (N–C=O). ^1^H NMR (400 MHz, DMSO-d6) δ 7.72 (d, J = 7.8 Hz, 1H), 7.66 (s, 1H, NH), 7.52 (d, J = 8.2 Hz, 2H), 7.32–7.07 (m, 7H), 6.77–6.70 (m, 2H), 6.27 (s, 1H), 2.23 (s, 3H); ^13^C NMR (100 MHz, DMSO-d6) δ 162.60, 146.89, 141.95, 141.40, 139.04, 137.98, 134.12, 129.37, 128.74, 128.39, 128.37, 127.55, 126.47, 123.98, 117.88, 115.84, 115.22, 73.02, 21.54 ppm; Anal. calcd. For C_21_H_17_BrN_2_O: C, 64.13; H, 4.36; and N, 7.12, Found: C, 64.17; H, 4.41; and N, 7.06.

#### 3-(4-ethyl phenyl)-2-(m-tolyl)-2,3-dihydroquinazolin-4(1H)-one (2k)

White solid; IR (KBr) ν (cm^−1^): 3293 (N–H), 1638 (N–C=O). ^1^H NMR (400 MHz, DMSO-d6) δ 7.73 (d, J = 7.8 Hz, 1H), 7.61 (s, 1H, NH), 7.26 (t, J = 7.7 Hz, 1H), 7.19–7.17 (m, 7H), 7.07 (d, J = 6.4 Hz, 1H), 6.76–6.69 (m, 2H); 6.20 (d, J = 2.5 Hz, 1H), 2.56 (q, J = 7.6 Hz, 2H), 2.23 (s, 3H), 1.16 (t, J = 7.4 Hz, 3H), ^13^C NMR (100 MHz, DMSO-d6) δ 162.67, 146.89, 141.95, 141.40, 139.04, 137.98, 134.12, 129.37, 128.74, 128.39, 128.37, 127.55, 126.47, 123.98, 117.88, 115.84, 115.22, 73.08, 28.13, 21.55, 16.01 ppm; Anal. calcd. For C_23_H_22_N_2_O: C, 80.67; H, 6.48; and N, 8.18, Found: C, 80.81; H, 6.48; and N, 8.19.

#### 3-(5-chloro-2-hydroxyphenyl)-2-(m-tolyl)-2,3-dihydroquinazolin-4(1H)-one (2l)

Brown solid; IR (KBr) ν (cm^−1^): 3344 (N–H), 3211 (O–H), 1614 (N–C=O). ^1^H NMR (400 MHz, DMSO-d6) δ 10.06 (s, 1H, OH), 7.71 (d, *J* = 7.8 Hz, 1H), 7.46–7.23 (m, 7H), 7.05 (dd, *J* = 8.7, 2.6 Hz, 1H), 6.93 (d, *J* = 2.7 Hz, 1H), 6.86–6.70 (m, 3H), 6.18 (s, 1H); ^13^C NMR (100 MHz, DMSO-d6) δ 162.85, 152.83, 147.93, 140.09, 134.16, 130.70, 129.21, 128.67, 128.53, 128.40, 127.77, 121.74, 117.94, 117.81, 115.03, 114.90, 73.02 ppm; Anal. calcd. For C_21_H_17_ClN_2_O_2_: C, 69.14; H, 4.70; and N, 7.68, Found: C, 69.18; H, 4.72; and N, 7.68.

#### 2-(5-(4-chlorophenyl)furan-2-yl)-3-(p-tolyl)-2,3-dihydroquinazolin-4(1H)-one (3a)

Off*-*white solid; IR (KBr) ν (cm^−1^): 3440 (N–H), 1682 (N–C=O). ^1^H NMR (400 MHz, DMSO-d6) δ 7.75 (dd, *J* = 7.6, 2.1 Hz, 1H), 7.69 (d, *J* = 3.1 Hz, 1H, NH), 7.54 (d, *J* = 8.6 Hz, 2H), 7.46 (d, *J* = 8.5 Hz, 2H), 7.32 (t, *J* = 8.4 Hz, 1H), 7.26 (d, *J* = 8.0 Hz, 2H), 7.21 (d, *J* = 8.1 Hz, 2H), 6.86 (dd, *J* = 5.9, 2.4 Hz, 2H), 6.78 (t, *J* = 7.5 Hz, 1H), 6.38 (d, *J* = 3.4 Hz, 1H), 6.27 (d, *J* = 3.0 Hz, 1H), 2.31 (s, 3H); ^13^C NMR (100 MHz, DMSO-d6) δ 162.30, 153.62, 152.04, 147.05, 138.49, 136.22, 134.07, 132.49, 129.70, 129.41, 129.22, 128.30, 126.79, 125.42, 118.42, 116.22, 115.42, 110.99, 107.55, 67.01, 21.04 ppm; MS (*m/z*): 415 (*M* +); Anal. calcd. For C_25_H_19_ClN_2_O_2_: C, 72.37; H, 4.62; and N, 6.75, Found: C, 72.37; H, 4.65; and N, 6.70.

#### 2-(5-(4-bromophenyl)furan-2-yl)-3-(p-tolyl)-2,3-dihydroquinazolin-4(1H)-one (3b)

Off*-*white solid; IR (KBr) ν (cm^−1^): 3315 (N–H), 1632 (N–C=O). ^1^H NMR (400 MHz, DMSO-d6) δ 7.75 (d, *J* = 7.8 Hz, 1H), 7.69 (d, *J* = 3.1 Hz, 1H, NH), 7.59 (d, J = 8.4 Hz, 1H), 7.48 (d, *J* = 8.6 Hz, 2H), 7.32 (t, *J* = 7.6 Hz, 1H), 7.26 (d, *J* = 8.0 Hz, 2H), 7.21 (d, *J* = 8.2 Hz, 2H), 6.90–6.83 (m, 2H), 6.78 (t, *J* = 7.6 Hz, 1H) 6.38 (d, *J* = 3.4 Hz, 1H), 6.27 (d, *J* = 2.9 Hz, 1H), 2.31 (s, 3H); ^13^C NMR (100 MHz, DMSO-d6) δ 162.32, 153.64, 152.09, 147.03, 138.48, 136.25, 134.09, 132.29, 129.72, 129.54, 128.31, 126.80, 125.69, 121.05, 118.46, 116.22, 115.43, 111.02, 107.62, 67.93, 21.04 ppm; MS (*m/z*): 460 (*M* +); Anal. calcd. For C_25_H_19_BrN_2_O_2_: C, 65.37; H, 4.17; and N, 6.10, Found: C, 65.37; H, 4.17; and N, 6.12.

#### 2-(5-(2,4-dichlorophenyl)furan-2-yl)-3-(p-tolyl)-2,3-dihydroquinazolin-4(1H)-one (3c)

Off*-*white solid; IR (KBr) ν (cm^−1^): 3277 (N–H), 1682 (N–C=O). ^1^H NMR (400 MHz, CDCl_3_) δ 8.05 (d, *J* = 7.9 Hz, 1H), 7.48 (d, *J* = 8.5 Hz, 1H), 7.43 (d, *J* = 2.2 Hz, 1H, NH), 7.38–7.31 (m, 2H), 7.27 (d, *J* = 8.0 Hz, 2H), 7.20 (d, *J* = 8.3 Hz, 3H), 6.99–6.90 (m, 2H), 6.74 (d, *J* = 8.1 Hz, 1H), 6.42 (d, *J* = 3.5 Hz, 1H), 6.06 (s, 1H), 2.36 (s, 3H); ^13^C NMR (100 MHz, CDCl_3_) δ 162.65, 152.30, 149.42, 148.57, 145.26, 138.03, 136.95, 135.75, 133.36, 130.56, 130.42, 129.85, 129.76, 128.89, 128.43, 127.27, 126.31, 119.91, 117.29, 115.19, 111.74, 110.83, 68.65, 21.09 ppm; MS (*m/z*): 450 (*M* +); Anal. calcd. For C_25_H_18_Cl_2_N_2_O_2_: C, 66.83; H, 4.04; and N, 6.23, Found: C, 66.83; H, 4.18; and N, 6.19.

#### 2-(5-(2-nitrophenyl)furan-2-yl)-3-(p-tolyl)-2,3-dihydroquinazolin-4(1H)-one (3d)

Off*-*white solid; IR (KBr) ν (cm^−1^): 3425 (N–H), 1681 (N–C=O). ^1^H NMR (400 MHz, DMSO-d6) δ 7.99–7.43 (m, 6H), 7.41–7.00 (m, 5H), 6.96–6.55 (m, 3H), 6.40 (s, 1H), 6.23 (s, 1H), 2.31 (s, 3H); ^13^C NMR (100 MHz, DMSO-d6)δ 162.07, 155.10, 148.07, 146.83, 138.43, 136.24, 135.55, 134.09, 130.82, 129.72, 129.35, 126.77, 124.94, 124.52, 122.95, 121.04, 118.45, 116.08, 115.39, 111.06, 110.93, 67.76, 21.05 ppm; MS (*m/z*): 426 (*M* +); Anal. calcd. For C_25_H_19_N_3_O_4_: C,70.58; H, 4.50; and N, 9.88, Found: C, 70.58; H, 4.69; and N, 9.78.

#### 2-(5-(2,4-dichlorophenyl)furan-2-yl)-3-(4-ethyl phenyl)-2,3-dihydroquinazolin-4(1H)-one (3e)

Off*-*white solid; IR (KBr) ν (cm^−1^): 3420 (N–H), 1640 (N–C=O). ^1^H NMR (400 MHz, CDCl_3_) δ 8.06 (dd, *J* = 7.9, 1.5 Hz, 1H), 7.49 (d, *J* = 8.5 Hz, 1H), 7.43 (d, *J* = 2.1 Hz, 1H, NH), 7.38–7.28 (m, 5H), 7.25–7.22 (m, 2H), 7.01–6.92 (m, 2H), 6.75 (d, *J* = 8.0 Hz, 1H), 6.44 (d, *J* = 3.5 Hz, 1H), 6.09 (s, 1H), 2.66 (q, *J* = 7.6 Hz, 2H), 1.25 (t, *J* = 7.6 Hz, 3H); ^13^C NMR (100 MHz, CDCl_3_)δ 162.51, 152.25, 149.51, 145.02, 143.22, 138.16, 133.74, 130.65, 130.46, 129.00, 128.60, 128.45, 127.28, 127.16, 126.29, 120.13, 117.21, 115.14, 111.76, 110.91, 68.70, 28.68, 15.48 ppm; MS (*m/z*): 464 (*M* +); Anal. calcd. For C_26_H_20_Cl_2_N_2_O_2_: C, 67.39; H, 4.35; and N, 6.05, Found: C, 67.42; H, 4.35; and N, 6.05.

#### 2-(5-(2,4-dichlorophenyl)furan-2-yl)-3-phenyl-2,3-dihydroquinazolin-4(1H)-one (3f)

Off*-*white solid; IR (KBr) ν (cm^−1^): 3277 (N–H), 1684 (N–C=O). ^1^H NMR (400 MHz, DMSO-d6) δ 7.82–7.73 (m, 2H), 7.68 (s, 1H, NH), 7.60 (d, *J* = 8.8 Hz, 1H), 6.79 (t, *J* = 7.4 Hz, 1H), 6.47 (s, 1H), 6.37 (s, 1H); ^13^C NMR (100 MHz, DMSO-d6) δ 162.29, 153.90, 148.57, 147.07, 140.99, 140.07, 134.19, 133.03, 130.62, 130.17, 129.27, 129.11, 128.39, 128.20, 127.42, 126.83, 118.53, 116.19, 115.51, 112.49, 110.98, 67.76 ppm; MS (*m/z*): 436 (*M* +); Anal. calcd. For C_24_H_16_Cl_2_N_2_O_2_: C, 66.22; H, 3.70; and N, 6.44, Found: C, 66.22; H, 3.78; and N, 6.42.

## Conclusion

In the current study, we presented a novel, eco-friendly, heterogeneous catalyst called sulfonated magnetic Spirulina. Combining this catalyst with water as a solvent showcased a powerful synergistic effect, enabling the successful synthesis of diverse dihydroquinazolinones at 60 °C. The sulfonic acid groups on the surface of Spirulina algae serve as active sites for catalytic reactions, while the magnetic properties of CoFe2O4 facilitate effortless separation and retrieval of the catalyst using an external magnetic field. Additionally, high yield of products in low reaction times, easy final product separation and purification are other advantages for this approach (Supplementary Informations S1).

### Supplementary Information

Supplementary Information.

## Data Availability

This published article and its supplementary information file include all data generated or analyzed during this study**.**

## References

[1] Dai H (2015). Environmental catalysis: A solution for the removal of atmospheric pollutants. Sci. Bull..

[2] Maddiboyina, B. *et al.* Food and drug industry applications of microalgae Spirulina platensis: A review. *J. Basic Microb.* (2023). 10.1002/jobm.202200704 36720046

[3] Al-Qahtani WH (2021). The value of blue-green algae (Spirulina platensis) as a nutritive supplement and toxicant against almond moth [Cadra cautella (Lepidoptera: Pyralidae)]. Plos one.

[4] Brito, A. d. F. *et al.* Spirulina platensis prevents oxidative stress and inflammation promoted by strength training in rats: Dose-response relation study. *Sci. Rep.* **10**, 6382 (2020). 10.1038/s41598-020-63272-5 PMC7156748 32286405

[5] Koli DK, Rudra SG, Bhowmik A, Pabbi S (2022). Nutritional, functional, textural and sensory evaluation of Spirulina enriched green pasta: A potential dietary and health supplement. Foods.

[6] Sadeghzadeh SM, Zhiani R, Emrani S (2018). Spirulina (Arthrospira) platensis Supported Ionic Liquid as a Catalyst for the Synthesis of 3-Aryl-2-oxazolidinones from Carbon Dioxide, Epoxide. Anilines. Catal. Lett..

[7] Karami-Osboo R, Ahmadpoor F, Nasrollahzadeh M, Maham M (2022). Polydopamine-coated magnetic Spirulina nanocomposite for efficient magnetic dispersive solid-phase extraction of aflatoxins in pistachio. Food Chem..

[8] Sharghi, H., Aali Hosseini, M., Aboonajmi, J. & Aberi, M. Use of vitamin B12 as a nontoxic and natural catalyst for the synthesis of benzoxazoles via catechols and primary amines in water under aerobic oxidation. *ACS Sustain. Chem. Eng.* **9**, 11163–11170 (2021).

[9] Clarke CJ, Tu W-C, Levers O, Brohl A, Hallett JP (2018). Green and sustainable solvents in chemical processes. Chem. Rev..

[10] Lee J (2020). Green-solvent-processable organic semiconductors and future directions for advanced organic electronics. J. Mater. Chem. A..

[11] Wu J (2014). Preparation of 2, 3-dihydroquinazolin-4 (1 H)-one derivatives in aqueous media with β-cyclodextrin-SO _3_ H as a recyclable catalyst. Green Chem..

[12] Shylesh S, Schünemann V, Thiel WR (2010). Magnetically separable nanocatalysts: Bridges between homogeneous and heterogeneous catalysis. Angew. Chem. Int. Ed..

[13] Zhang J (2018). Magnetically separable nanocatalyst with the Fe_3_O_4_ core and polydopamine-sandwiched Au nanocrystal shell. Langmuir.

[14] Adhikary J (2015). Development of an efficient magnetically separable nanocatalyst: theoretical approach on the role of the ligand backbone on epoxidation capability. RSC Adv..

[15] Safari J, Gandomi-Ravandi S (2013). Environmentally friendly synthesis of 2-aryl-2, 3-dihydroquinazolin-4 (1H)-ones by novel Co-CNTs as recoverable catalysts. C. R. Chim..

[16] Safaei-Ghomi J, Teymuri R (2021). A favourable ultrasound-assisted method for the combinatorial synthesis of 2, 3-dihydroquinazolin-4 (1H)-ones via CoAl_2_O_4_ spinel nanocrystal as an efficient catalyst. Iran. J. Catal..

[17] Badolato M, Aiello F, Neamati N (2018). 2, 3-Dihydroquinazolin-4 (1 H)-one as a privileged scaffold in drug design. RSC Adv..

[18] Mou J (2020). An aqueous facile synthesis of 2, 3-dihydroquinazolin-4 (1H)-one derivatives by reverse zinc oxide micelles as nanoreactor. Front. Chem..

[19] Yashwantrao G, Jejurkar VP, Kshatriya R, Saha S (2019). Solvent-free, mechanochemically scalable synthesis of 2, 3-dihydroquinazolin-4 (1H)-one using Brønsted acid catalyst. ACS Sustain. Chem. Eng..

[20] Khaleghi Abbasabadi M, Azarifar D (2020). β-Alanine-functionalized magnetic graphene oxide quantum dots: an efficient and recyclable heterogeneous basic catalyst for the synthesis of 1H-pyrazolo [1, 2-b] phthalazine-5, 10-dione and 2, 3-dihydroquinazolin-4 (1H)-one derivatives. Appl. Organomet. Chem..

[21] Sivaguru P, Parameswaran K, Lalitha A (2017). Antioxidant, anticancer and electrochemical redox properties of new bis (2, 3-dihydroquinazolin-4 (1 H)-one) derivatives. Mol. Divers..

[22] Maleki A, Kari T, Aghaei M (2017). Fe_3_O_4_@ SiO_2_@ TiO_2_-OSO_3_ H: An efficient hierarchical nanocatalyst for the organic quinazolines syntheses. J. Porous Mat..

[23] Liu C-H, Wang Q, Xu Z, Li D, Zheng Y (2022). 5, 5’-Indigodisulfonic acid as an efficient catalyst for the synthesis of 2, 3-dihydroquinazolinone derivatives. Synth. Commun..

[24] Rostamizadeh S, Amani AM, Mahdavinia GH, Sepehrian H, Ebrahimi S (2010). Synthesis of some novel 2-aryl-substituted 2, 3-dihydroquinazolin-4 (1H)-ones under solvent-free conditions using MCM-41-SO_3_H as a highly efficient sulfonic acid. Synth..

[25] Solgi S, Ghorbani-Vaghei R, Alavinia S, Izadkhah V (2022). Preparation and application of highly efficient and reusable nanomagnetic catalyst supported with sulfonated-hexamethylenetetramine for synthesis of 2, 3-dihydroquinazolin-4 (1 H)-ones. Polycyclic. Aromat. Compd..

[26] Kulangiappar K, Anbukulandainathan M, Raju T (2014). Nuclear versus side-chain bromination of 4-methoxy toluene by an electrochemical method. Synth. Commun..

[27] Ahmadian F (2019). Synthesis of pyrazol-quinazolinones and 2, 3-dihydroquinazolin-4 (1H)-ones using CoAl2O4 nanoparticles as heterogeneous catalyst. J. Iran. Chem. Soc..

[28] Keikhosravi, N. *et al.* Solvent-free synthesis and antimicrobial activity of dihydroquinazolinone derivatives. *J. Med. Chem. Sci.* (2022).

[29] Kohli S, Rathee G, Hooda S, Chandra R (2023). An efficient approach for the green synthesis of biologically active 2, 3-dihydroquinazolin-4 (1 H)-ones using a magnetic EDTA coated copper based nanocomposite. RSC Adv..

[30] Alinezhad H, Soleymani E, Zare M (2017). Facile method for the synthesis of 2, 3-dihydroquinazolin-4 (1 H)-ones catalyzed by SiO_2_–H_3_ PW_12_ O_40_ in water. Res. Chem. Intermediat.

[31] Safaei-Ghomi J, Teymuri R, Bakhtiari A (2019). Co-aminobenzamid@ Al-SBA-15: A favorable catalyst in synthesis of 2, 3-dihydroquinazolin-4 (1H)-ones. BMC Chem..

[32] Kancherla M, Katlakanti MR, Seku K, Badathala V (2019). Boric acid supported on montmorillonites as catalysts for synthesis of 2, 3-dihydroquinazolin-4 (1H)-ones. Iran. J. Chem. Chem. Eng..

[33] Saka C, Kaya M, Bekiroğullari M (2020). Spirulina Platensis microalgae strain modified with phosphoric acid as a novel support material for Co–B catalysts: Its application to hydrogen production. Int. J. Hydrogen Energ..

[34] Soni RA, Sudhakar K, Rana R (2021). Biochemical and thermal analysis of spirulina biomass through FTIR, TGA. CHN. Energy Eng..

[35] Yang JC, Yin X-B (2017). CoFe_2_O_4_@ MIL-100 (Fe) hybrid magnetic nanoparticles exhibit fast and selective adsorption of arsenic with high adsorption capacity. Sci. Rep..

[36] Nurazzi NM (2021). Thermogravimetric analysis properties of cellulosic natural fiber polymer composites: a review on influence of chemical treatments. Polymers.

[37] Wang M, Zhang TT, Liang Y, Gao JJ (2012). Efficient synthesis of mono-and disubstituted 2, 3-dihydroquinazolin-4 (1 H)-ones using copper benzenesulfonate as a reusable catalyst in aqueous solution. Monatsh. Chem..

[38] Bodaghifard MA, Safari S (2021). Cu (II) complex-decorated hybrid nanomaterial: A retrievable catalyst for green synthesis of 2, 3-dihydroquinazolin-4 (1 H)-ones. J. Coord. Chem..

[39] Razavi N, Akhlaghinia B (2016). Hydroxyapatite nanoparticles (HAP NPs): A green and efficient heterogeneous catalyst for three-component one-pot synthesis of 2, 3-dihydroquinazolin-4 (1 H)-one derivatives in aqueous media. New. J. Chem..

[40] Zhang ZH, Lu H-Y, Yang S-H, Gao J-W (2010). Synthesis of 2, 3-dihydroquinazolin-4 (1 H)-ones by three-component coupling of isatoic anhydride, amines, and aldehydes catalyzed by magnetic Fe_3_O_4_ nanoparticles in water. J. Comb. Chem..

[41] Dabiri M, Salehi P, Otokesh S, Baghbanzadeh M, Kozehgary G, Mohammadi A-A (2005). Efficient synthesis of mono-and disubstituted 2, 3-dihydroquinazolin-4 (1H)-ones using KAl (SO_4_)_2_· 12H_2_O as a reusable catalyst in water and ethanol. Tetrahedron Lett..

