# Supplementary material for: Sulfonated magnetic spirulina nanobiomaterial as a novel and environmentally friendly catalyst for the synthesis of dihydroquinazolin-4(1H)-ones in aqueous medium
Source: Sci Rep. 2024 Jan 27;14:2296. doi: 10.1038/s41598-024-52749-2 (PMC10821933; doi:10.1038/s41598-024-52749-2)
Supplement: Supplementary file 1 — Supplementary Information. [file 41598_2024_52749_MOESM1_ESM.docx]

**Sulfonated Magnetic Spirulina Nanobiomaterial as a Novel and Environmentally Friendly Catalyst for the Synthesis of Dihydroquinazolin-4(1H)-ones in Aqueous Medium**

Elahe Mashhadi^1^, Javad Safaei-Ghomi*^1^

^1^Department of Organic Chemistry, Faculty of Chemistry, University of Kashan, Kashan, I. R. Iran,

*E-mail address: [safaei@kashanu.ac.ir](mailto:safaei@kashanu.ac.ir).

**2,3-diphenyl-2,3-dihydroquinazolin-4(1H)-one** (2a)

White solid; 98% yield; IR (KBr) ν (cm^−1^): 3293 (N–H), 1629 (N–C = O). ^1^H NMR (500 MHz, DMSO-d6) δ 7.74 (d, J = 7.6 Hz, 1H), 7.67 (d, J = 2.7 Hz, 1H, NH), 7.40-7.24 (m, 10H), 7.19 (t, J = 7.2 Hz,1H), 6.78 (d, J = 8.1 Hz, 1H), 6.72 (t, J = 7.5 Hz, 1H), 6.30 (d, J = 2.7 Hz, 1H); ^13^C NMR (100 MHz, DMSO-d6) δ 166.63, 150.01, 144.15, 144.03,137.11, 131.96, 131.72, 131.55, 130.01, 129.58, 129.36, 120.88, 118.69, 117.85, 75.97 ppm;


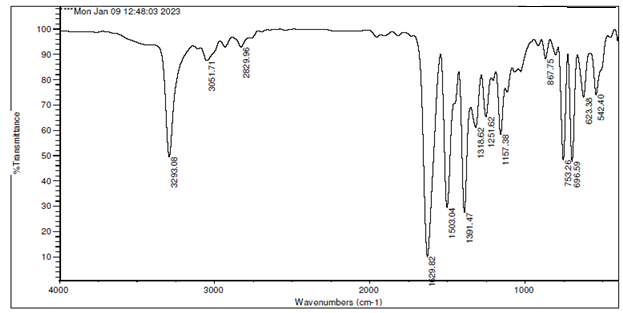


Figure S1: FT-IR spectrum of compound 2a


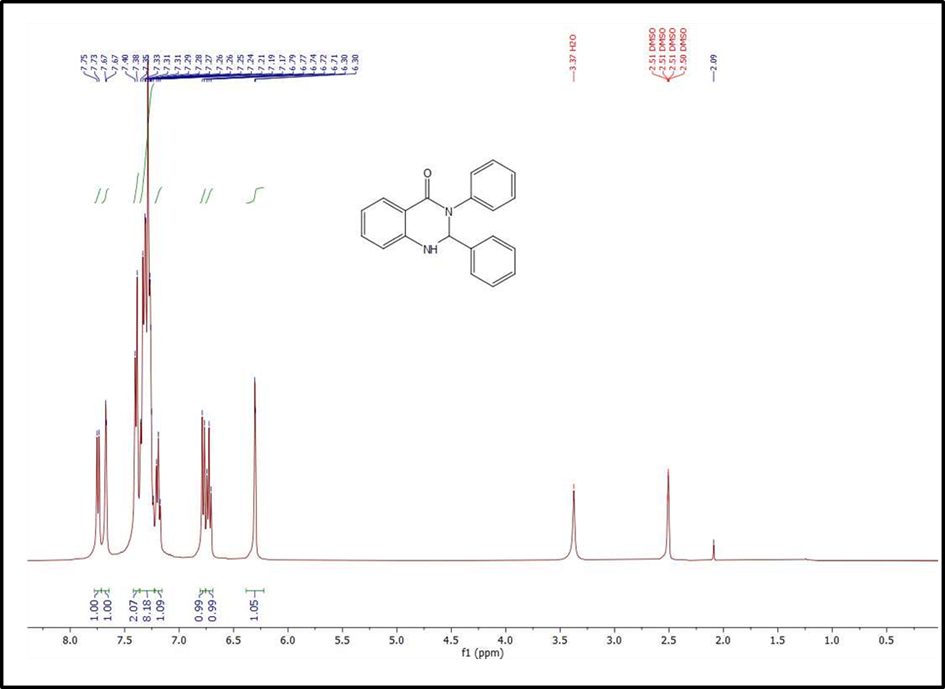


Figure S2: ^1^H NMR spectrum of compound 2a

**2-(4-chlorophenyl)-3-phenyl-2,3-dihydroquinazolin-4(1H)-one** (2b)


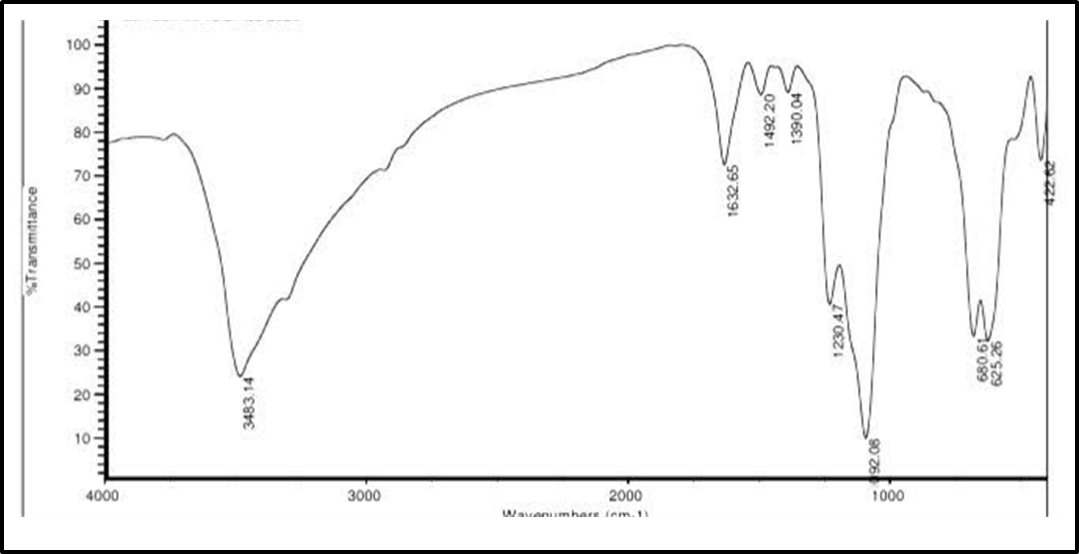
White solid; IR (KBr) ν (cm^−1^): 3483 (N–H), 1632 (N–C = O).^1^H NMR (400 MHz, DMSO-d6) *δ* 7.74-7.67 (m, 2H), 7.39-7.18 (m, 10H), 6.78-6.71 (m, 2H), 6.33 (s, 1H); ^13^C NMR (100 MHz, DMSO-d6) *δ* 165.44, 149.63, 143.88, 142.95, 137.23, 136.26, 132.04, 131.87, 132.03, 129.61, 129.41, 121.10, 118.62, 118.20, 75.32 ppm;

Figure S3: FT-IR spectrum of compound 2b


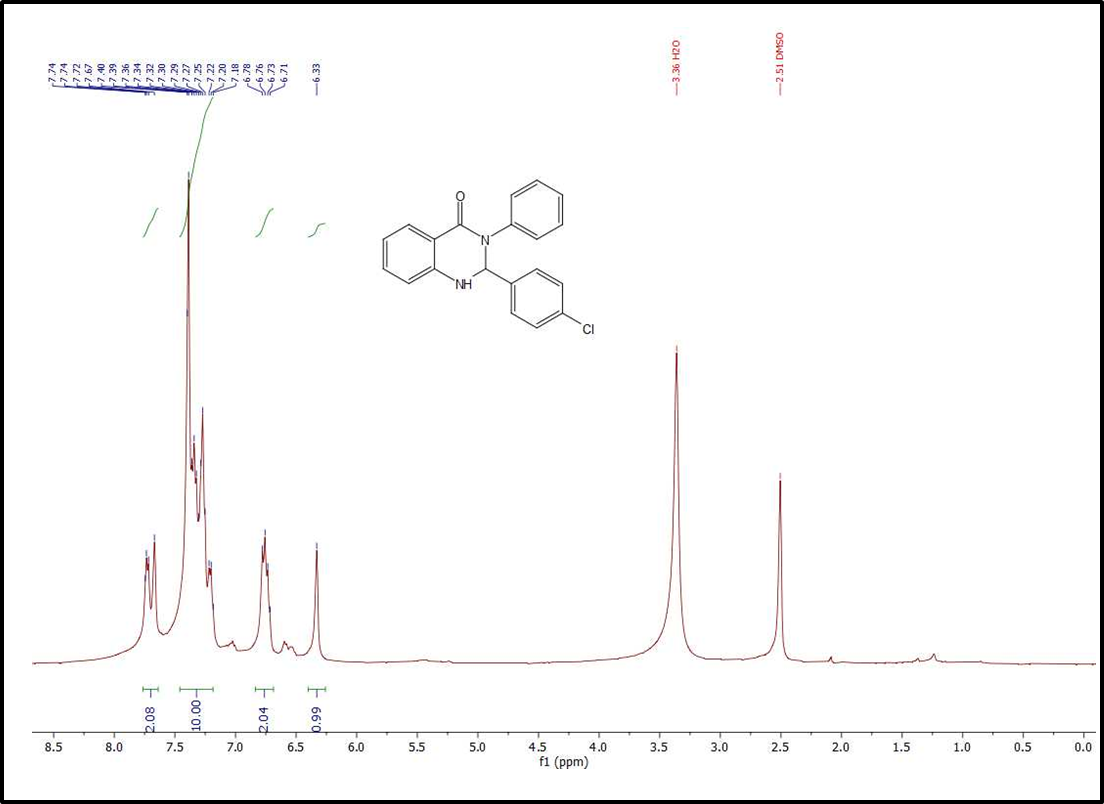


Figure S4: ^1^H NMR spectrum of compound 2b

**2-(4-bromophenyl)-3-phenyl-2,3-dihydroquinazolin-4(1H)-one** (2c)


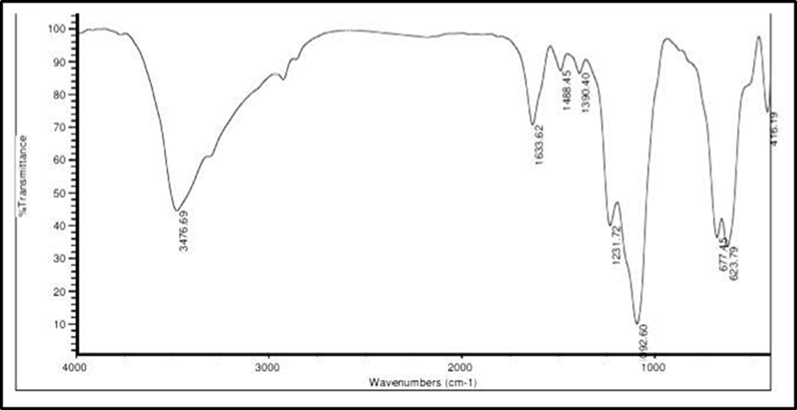
White solid; IR (KBr) ν (cm^−1^): 3476 (N–H), 1633 (N–C = O). ^1^H NMR (400 MHz, DMSO-d6) δ 7.72 (d, J = 7.8 Hz, 1H), 7.67 (s, 1H, NH), 7.56 – 7.49 (m, 2H), 7.19-7.37 (m, 8H), 6.72-6.78 (m, 2H), 6.31 (d, J = 2.6 Hz, 1H); ^13^C NMR (100 MHz, DMSO-d6) δ 162.63, 146.85, 141.09, 140.57, 134.35, 131.80, 129.31, 129.17, 128.49, 126.70, 126.61, 122.01, 118.21, 115.77, 115.34, 72.46 ppm;

Figure S5: FT-IR spectrum of compound 2c


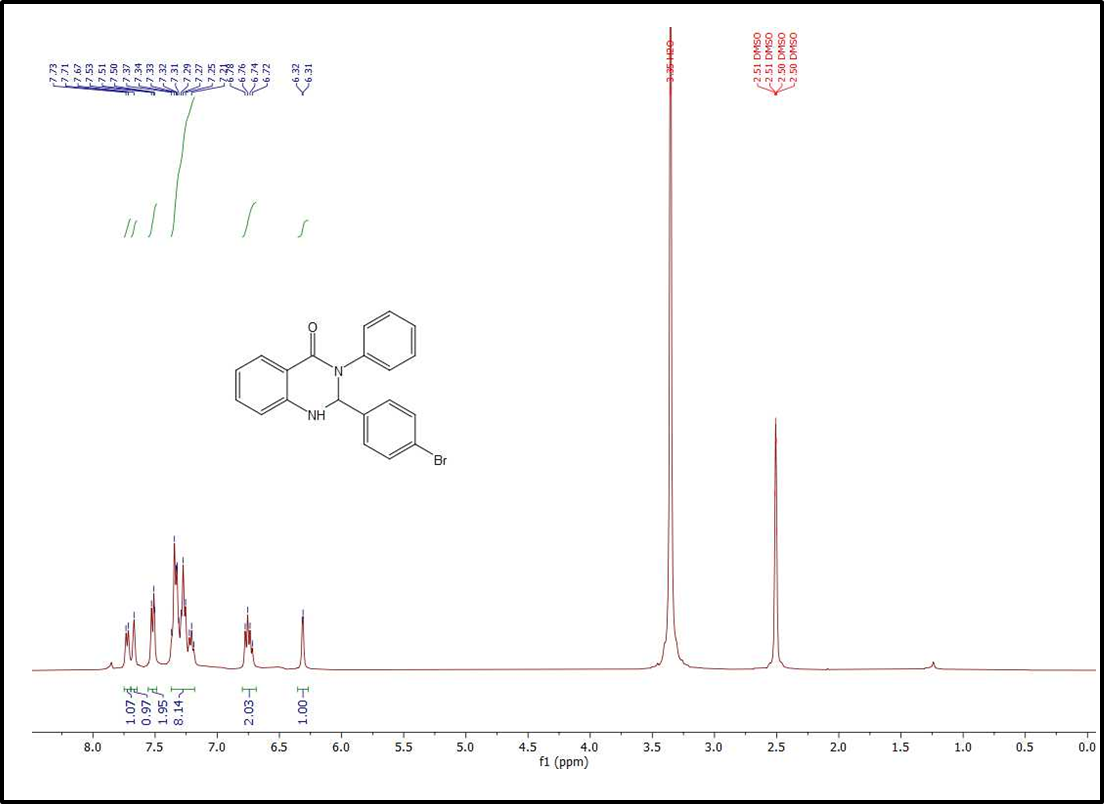


Figure S6: : 1H NMR spectrum of compound 2c

**3-phenyl-2-(p-tolyl)-2,3-dihydroquinazolin-4(1H)-one** (2d)

White solid; IR (KBr) ν (cm^−1^): 3298 (N–H), 1632 (N–C = O). ^1^H NMR (400 MHz, DMSO-d6) δ 7.73 (d, J = 7.8 Hz, 1H), 7.61 (d, J = 2.7 Hz, 1H, NH), 7.35-7.25 (m, 7H), 7.19 (t, J = 7.2 Hz, 1H), 7.10 (d, J = 7.8, Hz, 2H), 6.75 (d, J = 8.2 Hz, 1H), 6.71 (t, J = 7.5 Hz, 1H), 6.24 (d, J = 2.7 Hz, 1H), 2.22 (s, 3H); ^13^C NMR (100 MHz, DMSO-d6) δ 165.61, 149.91, 144.22, 141.14, 140.90, 137.05, 132.26, 131.83, 131.22, 129.82, 128.94, 129.27, 121.09, 118.74, 118.14, 75.77, 23.95 ppm;


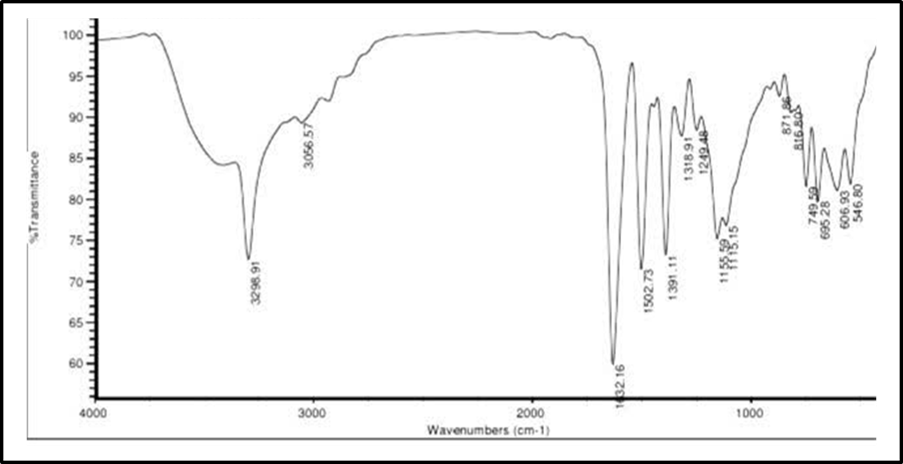


Figure S6: ^1^H NMR spectrum of compound 4c

Figure S6: ^1^H NMR spectrum of compound 4c

Figure S7: FT-IR spectrum of compound 2d


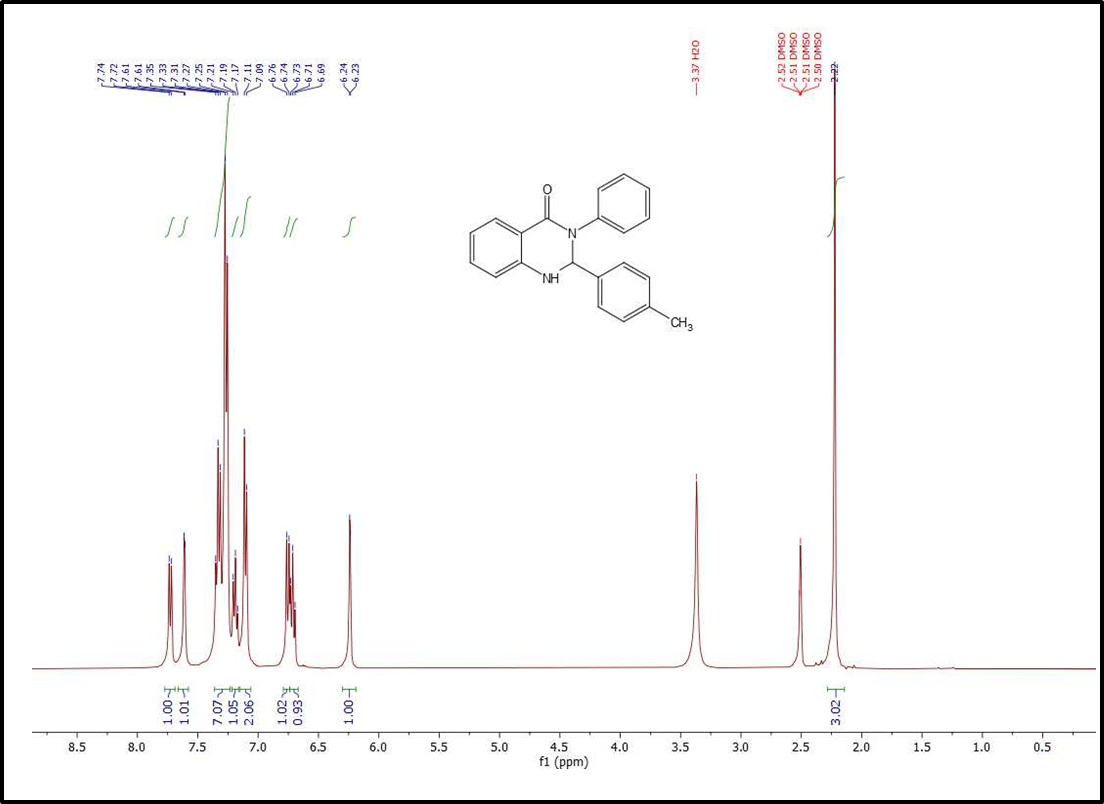


Figure S8: : ^1^H NMR spectrum of compound 2d

**2-(4-methoxyphenyl)-3-phenyl-2,3-dihydroquinazolin-4(1H)-one** (2e)


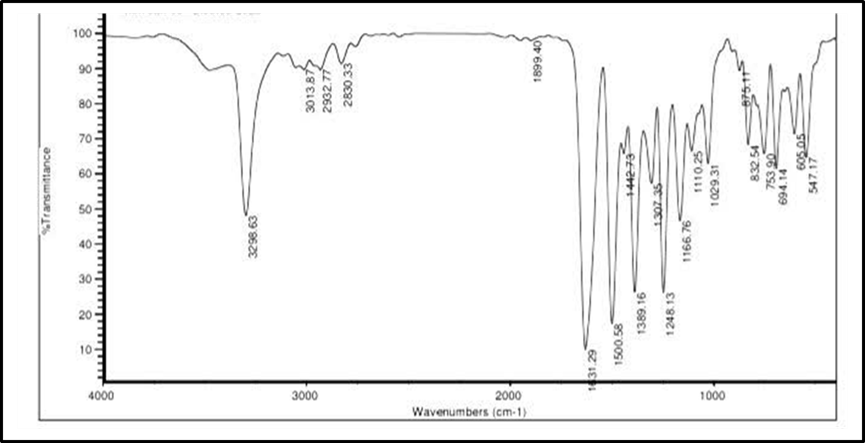
White solid; IR (KBr) ν (cm^−1^): 3298 (N–H), 1631 (N–C = O). ^1^H NMR (400 MHz, DMSO-d6) δ 7.73 (d, J = 7.8 Hz, 1H), 7.57 (s, 1H, NH), 7.34-7.25 (m, 7H), 7.19 (t, J = 7.4 Hz, 1H), 6.85 (d, J = 8.2 Hz, 2H), 6.76 (d, J = 8.1 Hz, 1H), 6.72 (t, J = 7.5 Hz, 1H), 6.23 (s, 1H), 3.69 (s, 3H); ^13^C NMR (100 MHz, DMSO-d6) δ 163.2, 156.6, 147.2, 141.2, 134.0, 133.9, 128.8, 127.2, 127.0, 117.8, 115.6, 115.4, 115.0, 114.1, 72.9, 55.4 ppm;

Figure S9: FT-IR spectrum of compound 2e


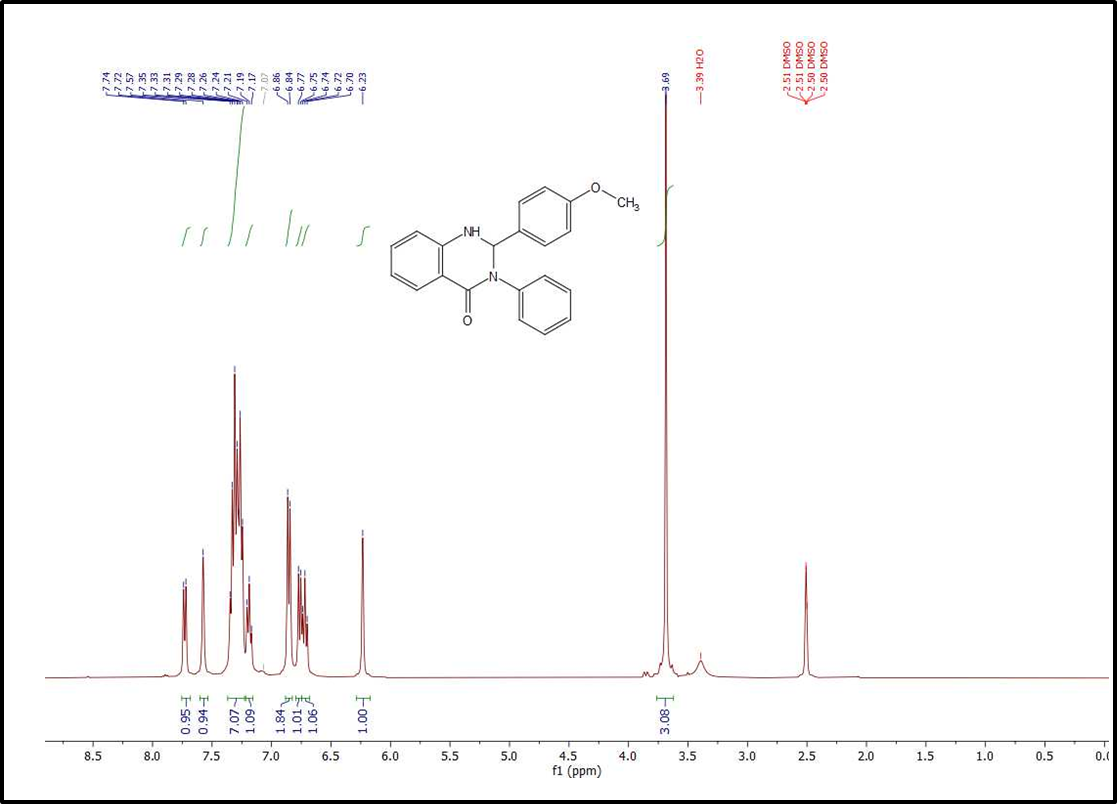


Figure S10: : ^1^H NMR spectrum of compound 2e

**2-(4-nitrophenyl)-3-(p-tolyl)-2,3-dihydroquinazolin-4(1H)-one** (2f)


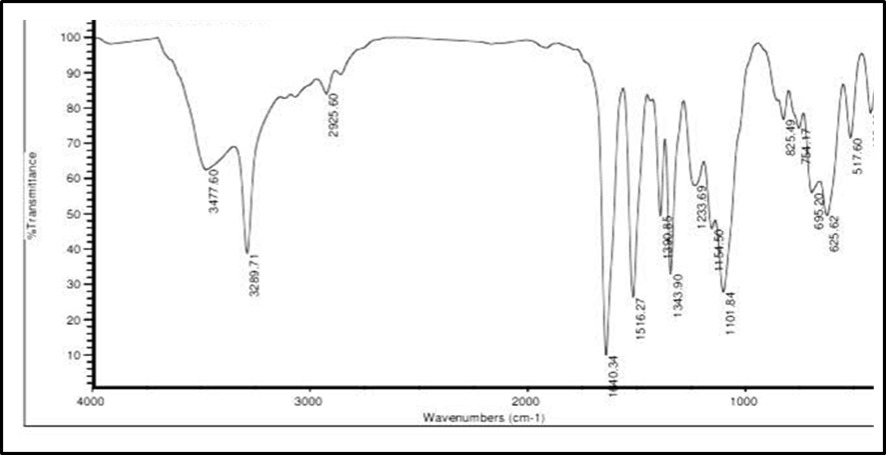
White solid; IR (KBr) ν (cm^−1^): 3289 (N–H), 1640 (N–C = O). ^1^H NMR (400 MHz, DMSO-d6) δ 8.19 (d, J = 8.3 Hz, 2H), 7.73-7.77 (m, 2H), 7.65 (d, J = 8.3 Hz, 2H), 7.29 (t, J = 7.6 Hz, 1H), 7.20-7.14 (m, 4H), 6.78-6.72 (m, 2H), 6.45 (d, J = 2.8 Hz, 1H), 2.27 (s, 3H); ^13^C NMR (100 MHz, DMSO-d6) δ 160.5, 149.1, 148.2, 141.0, 136.3, 132.5, 131.6, 130.2, 129.8, 129.2, 129.1, 126.7, 124.0, 119.5,119.0, 70.2, 20.4 ppm;

Figure S11: FT-IR spectrum of compound 2f


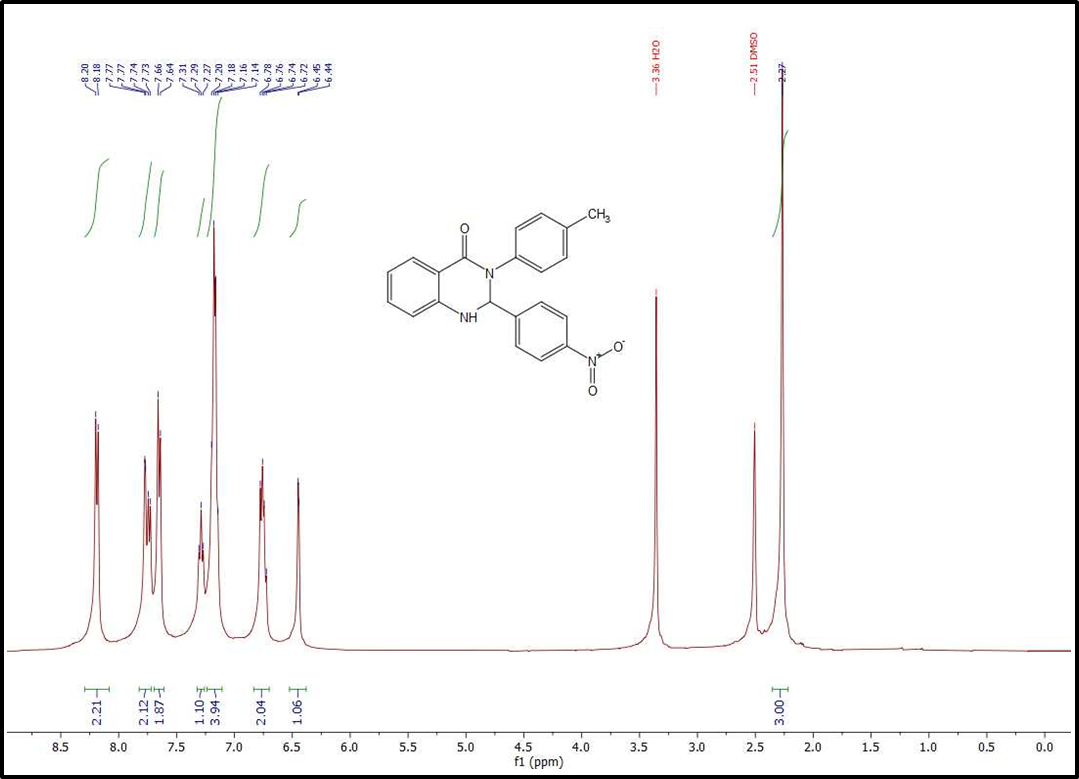


Figure S12: : ^1^H NMR spectrum of compound 2f

**2-(4-chlorophenyl)-3-(p-tolyl)-2,3-dihydroquinazolin-4(1H)-one** (2g)

White solid; IR (KBr) ν (cm^−1^): 3296 (N–H), 1632 (N–C = O). ^1^H NMR (400 MHz, DMSO-d6) δ 7.73 (d, J = 7.8 Hz, 1H), 7.61 (s, 1H, NH), 7.43 – 7.32 (m, 4H), 7.32 – 7.25 (m, 1H), 7.14 (br s, 4H), 6.80 – 6.69 (m, 2H), 6.28 (d, J = 2.6 Hz, 1H), 2.26 (s, 3H); ^13^C NMR (100 MHz, DMSO-d6) δ 162.3, 146.6, 139.5, 137.7, 137.3, 133.8, 130.02, 128.9, 128.4, 128.3, 127.9, 126.5, 117.48, 115.03, 114.7, 72.25, 20.59 ppm;


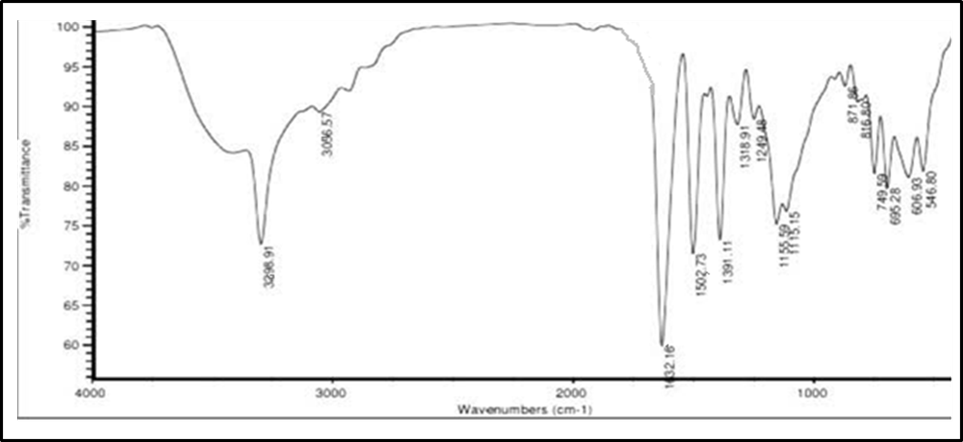


Figure S13: FT-IR spectrum of compound 2g

Figure S14: : ^1^H NMR spectrum of compound 2g

Figure S13: FT-IR spectrum of compound 2g


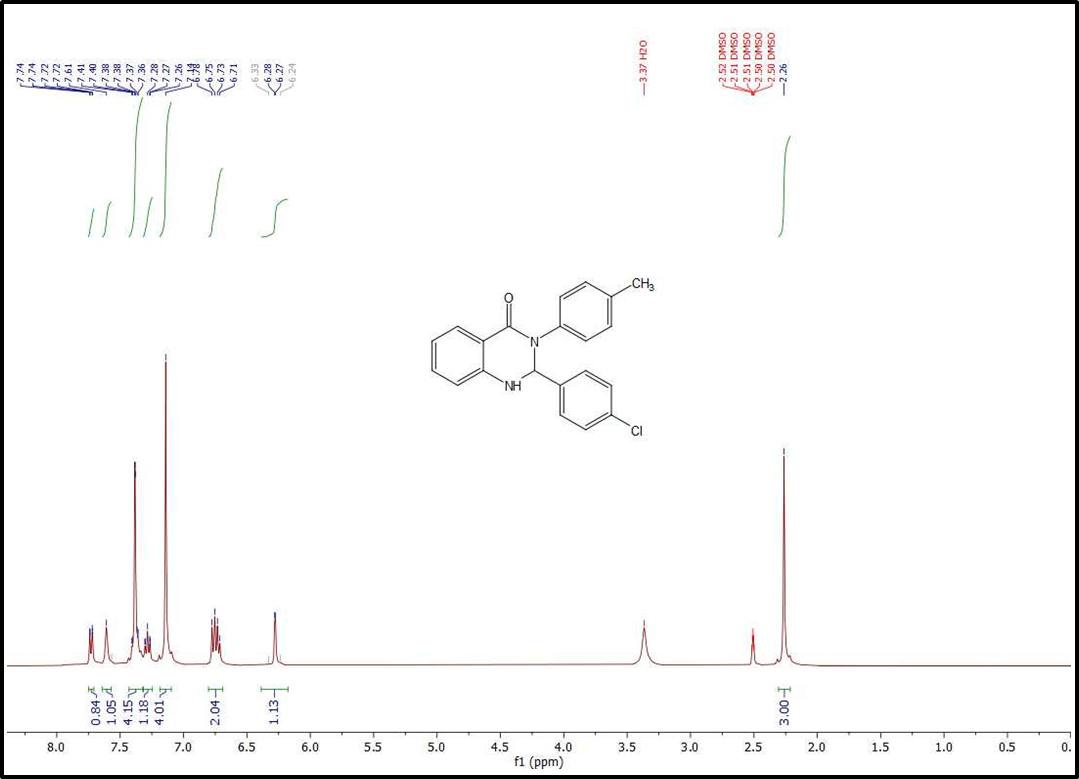
**2-(4-methoxyphenyl)-3-(p-tolyl)-2,3-dihydroquinazolin-4(1H)-one** (2h)


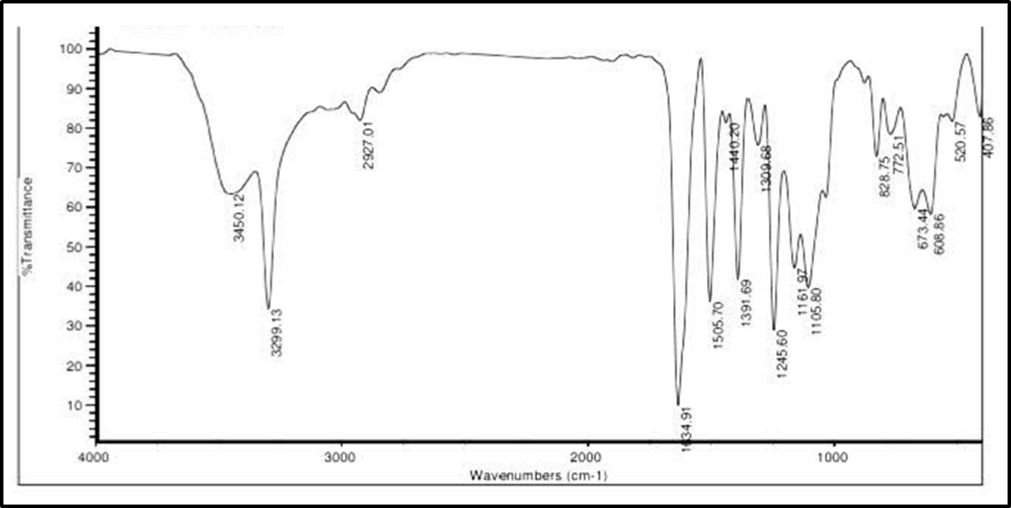
White solid; IR (KBr) ν (cm^−1^): 3299 (N–H), 1634 (N–C = O). ^1^H NMR (400 MHz, DMSO-d6) δ 7.71 (d, J = 7.8 Hz, 1H), 7.53 (d, J = 2.6 Hz, 1H, NH), 7.28 (m, 3H), 7.12 (br s, 4H), 6.85 (dd, J = 9.0, 2.5 Hz, 2H), 6.78 – 6.67 (m, 2H), 6.18 (d, J = 2.5 Hz, 1H), 3.68 (s, 3H), 2.26 (s, 3H); ^13^C NMR (100 MHz, DMSO-d6) δ 171.3, 158.7, 146.8, 144.2, 141.3, 137.6, 136.8, 132.5, 129.6, 128.8, 128.0, 127.5, 125.3, 123.7, 123.5, 122.5, 115.3, 110.1, 72.4, 55.4, 20.6 ppm;

Figure S15: FT-IR spectrum of compound 2h


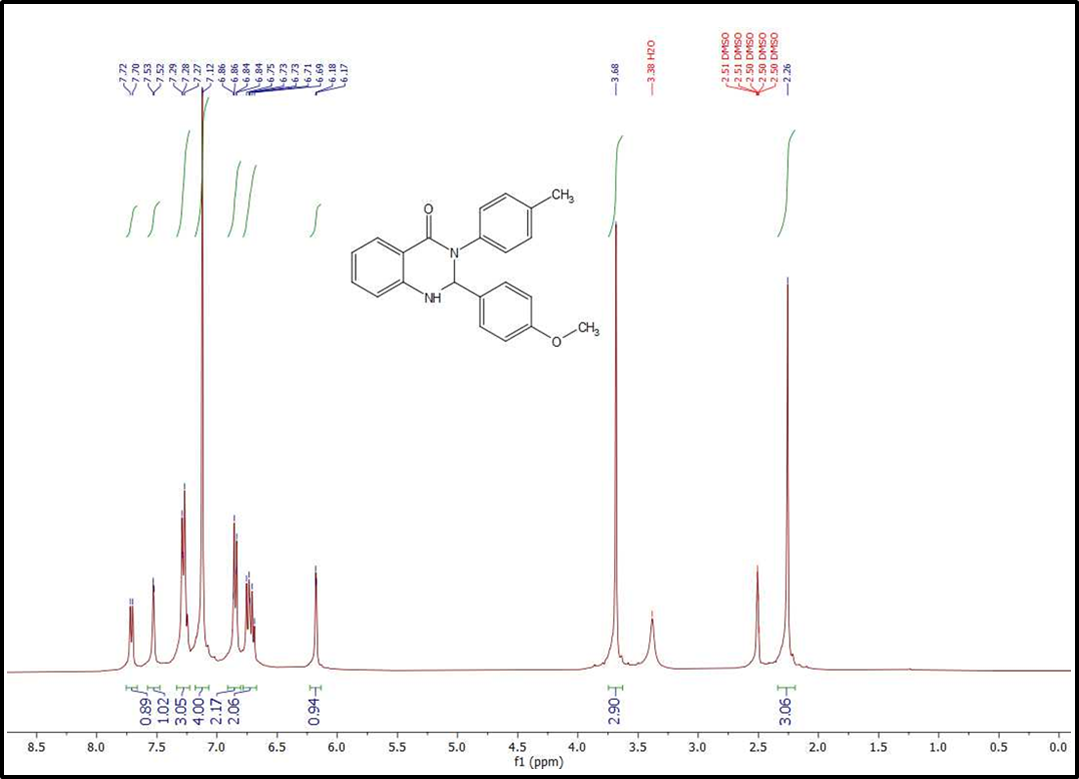


Figure S16: ^1^H NMR spectrum of compound 2h

**3-phenyl-2-(m-tolyl)-2,3-dihydroquinazolin-4(1H)-one** (2i)

White solid; IR (KBr) ν (cm^−1^): 3300 (N–H), 1634 (N–C = O). ^1^H NMR (400 MHz, DMSO-d6) δ 7.73 (dd, J = 7.8, 1.6 Hz, 1H), 7.63 (d, J = 2.8 Hz, 1H, NH), 7.35-7.32 (m, 2H), 7.29-7.25 (m, 3H), 7.21-7.18 (m, 4H), 7.11 – 7.04 (m, 1H), 6.76 (d, J = 8.1 Hz, 1H), 6.72 (t, J = 7.5 Hz, 1H), 6.24 (d, J = 2.7 Hz, 1H), 2.23 (s, 3H); ^13^C NMR (100 MHz, DMSO-d6) δ 161.1, 145.6, 144.3, 139.1, 138.2, 132.9, 130.2, 128.9, 128.4, 128.3, 128.0, 127.5, 122.9, 117.2, 115.1, 85.6, 21.06 ppm;


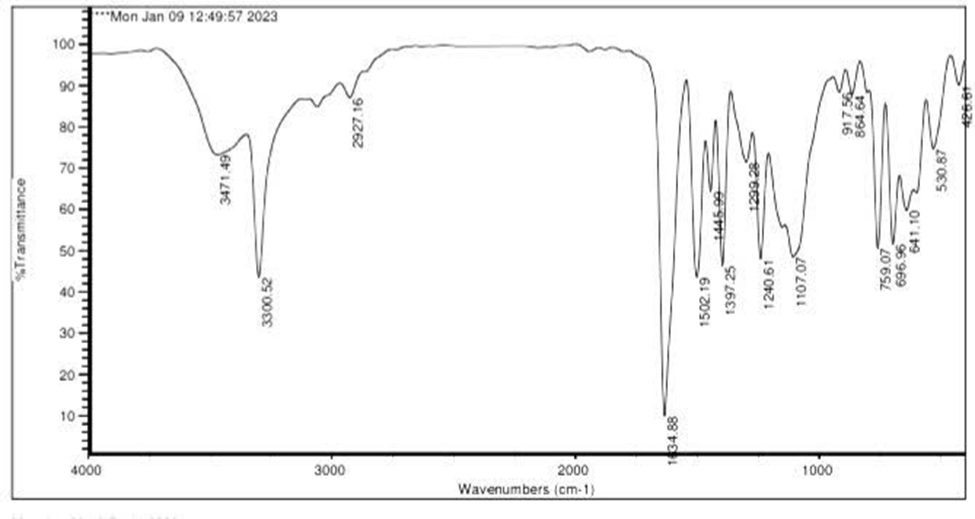


Figure S17: FT-IR spectrum of compound 2i


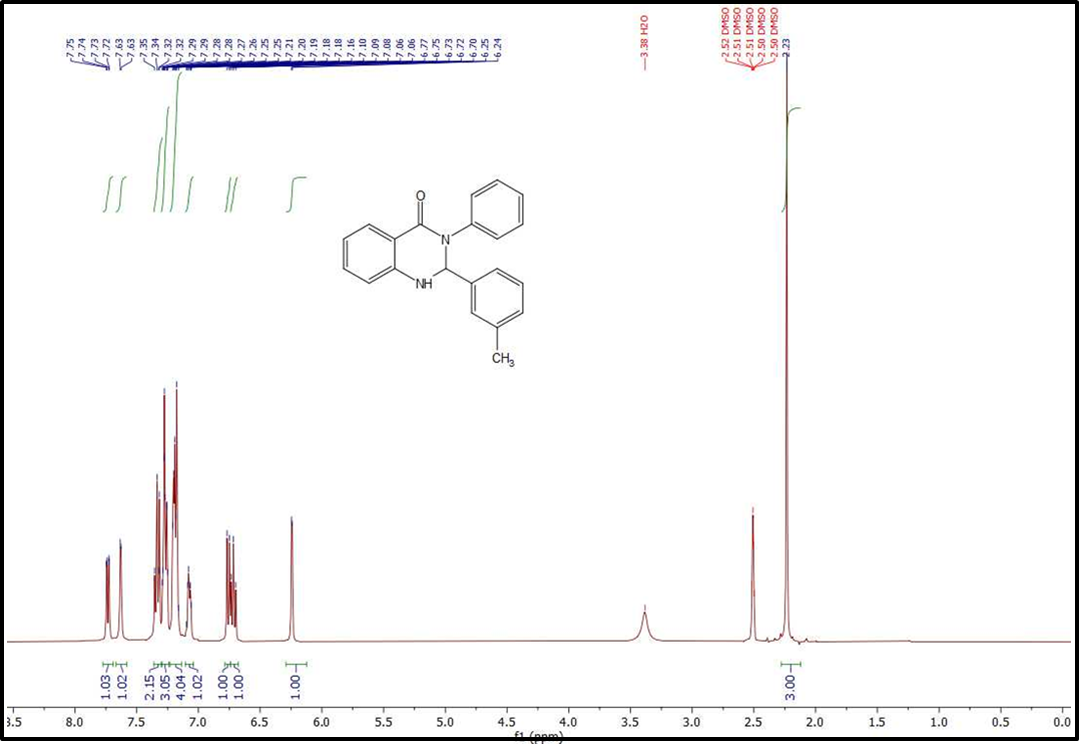


Figure S18: ^1^H NMR spectrum of compound 2i

**3-(4-bromophenyl)-2-(m-tolyl)-2,3-dihydroquinazolin-4(1H)-one** (2j)

White solid; IR (KBr) ν (cm^−1^): 3301 (N–H), 1632 (N–C = O). ^1^H NMR (400 MHz, DMSO-d6) δ 7.72 (d, J = 7.8 Hz, 1H), 7.66 (s, 1H, NH), 7.52 (d, J = 8.2 Hz, 2H), 7.32 – 7.07 (m, 7H), 6.77 – 6.70 (m, 2H), 6.27 (s, 1H), 2.23 (s, 3H); ^13^C NMR (100 MHz, DMSO-d6) δ 162.60, 146.89, 141.95, 141.40, 139.04, 137.98, 134.12, 129.37, 128.74, 128.39, 128.37, 127.55, 126.47, 123.98, 117.88, 115.84, 115.22, 73.02, 21.54 ppm; Anal. calcd. For C_21_H_17_BrN_2_O: C, 64.13; H, 4.36; and N, 7.12, Found: C, 64.17; H, 4.41; and N, 7.06

Figure S19: FT-IR spectrum of compound 2j


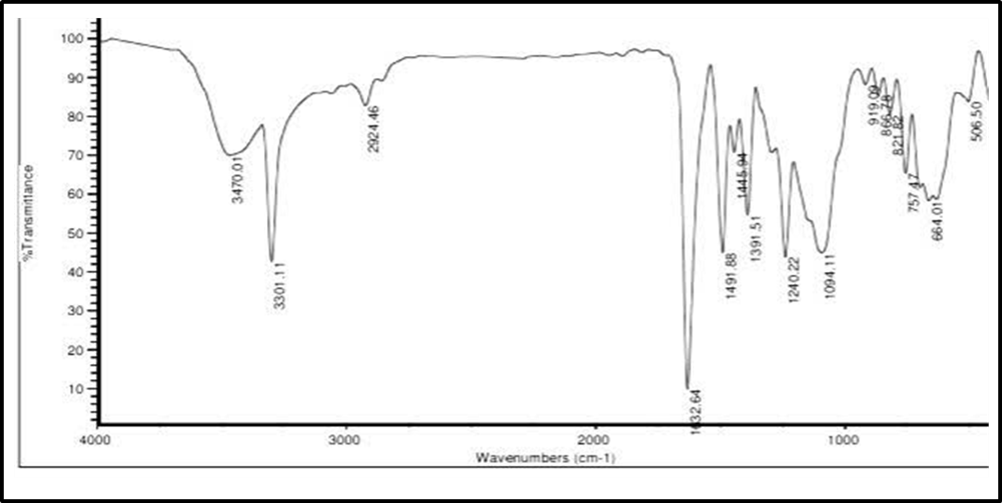


**
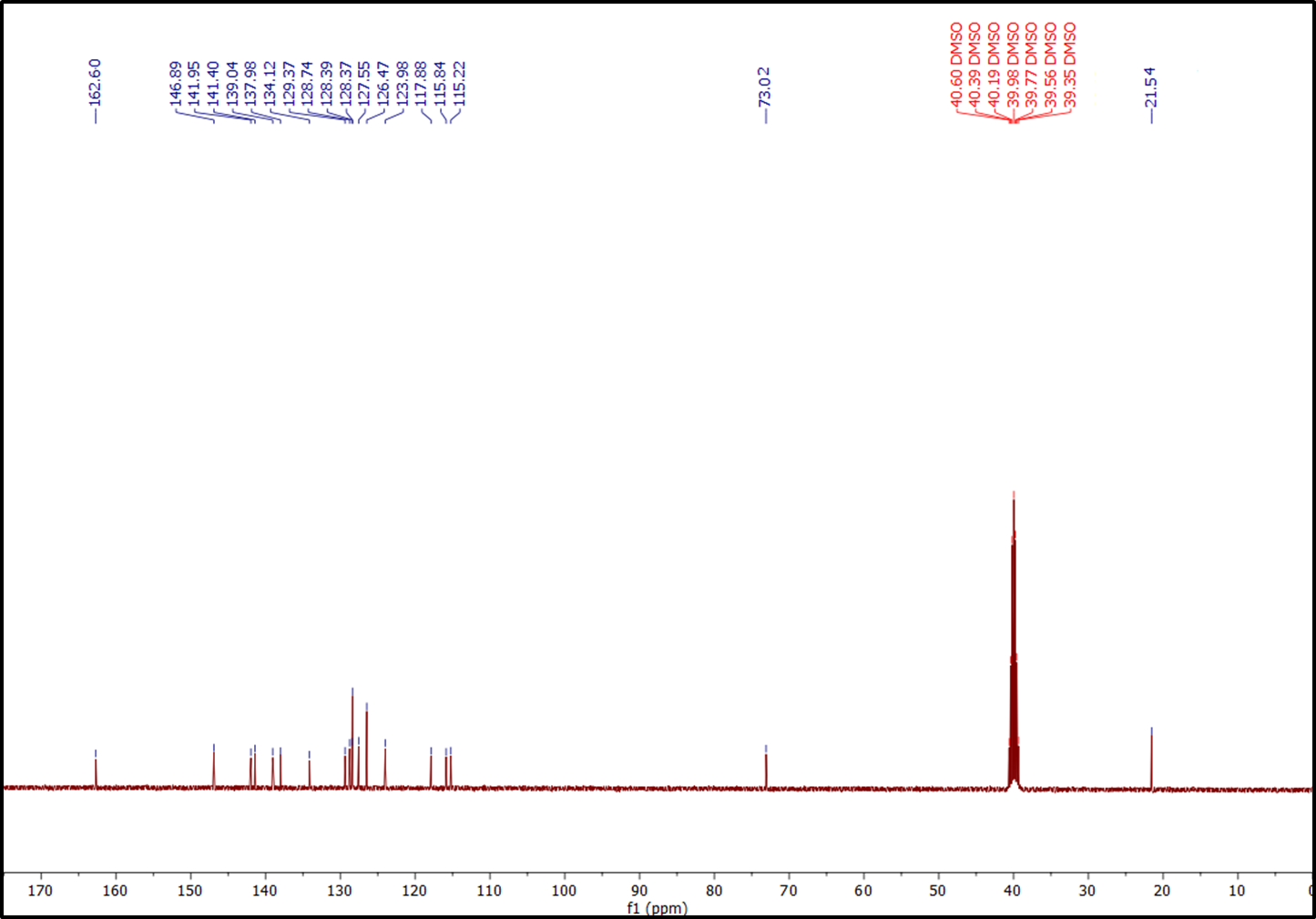

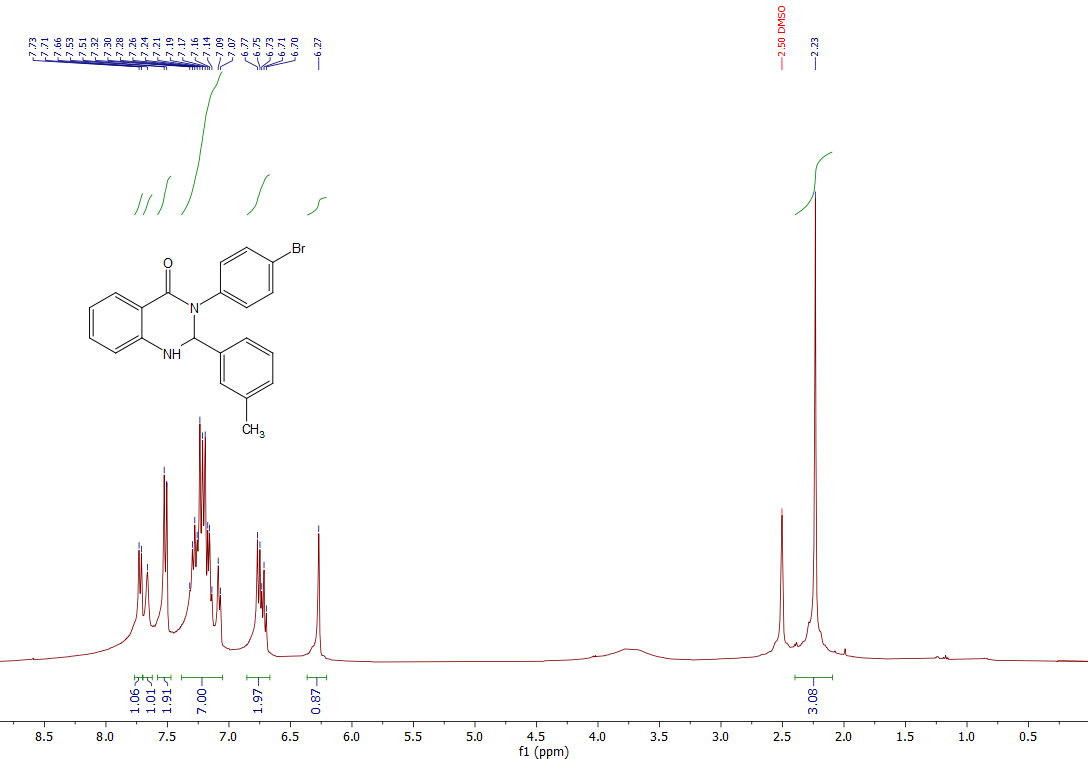
**

Figure S20: ^1^H NMR spectrum of compound 2j

Figure S21: ^13^C NMR spectrum of compound 2j

**3-(4-ethylphenyl)-2-(m-tolyl)-2,3-dihydroquinazolin-4(1H)-one (2k)**

White solid; IR (KBr) ν (cm^−1^): 3293 (N–H), 1638 (N–C = O). ^1^H NMR (400 MHz, DMSO-d6) δ 7.73 (d, J = 7.8 Hz, 1H), 7.61 (s, 1H, NH), 7.26 (t, J = 7.7 Hz, 1H), 7.19-7.17 (m, 7H), 7.07 (d, J = 6.4 Hz, 1H), 6.76-6.69 (m, 2H); 6.20 (d, J = 2.5 Hz, 1H), 2.56 (q, J = 7.6 Hz, 2H), 2.23 (s, 3H), 1.16 (t, J = 7.4 Hz, 3H), ^13^C NMR (100 MHz, DMSO-d6) δ 162.67, 146.89, 141.95, 141.40, 139.04, 137.98, 134.12, 129.37, 128.74, 128.39, 128.37, 127.55, 126.47, 123.98, 117.88, 115.84, 115.22, 73.08, 28.13, 21.55, 16.01 ppm; Anal. calcd. For C_23_H_22_N_2_O: C, 80.67; H, 6.48; and N, 8.18, Found: C, 80.81; H, 6.48; and N, 8.19


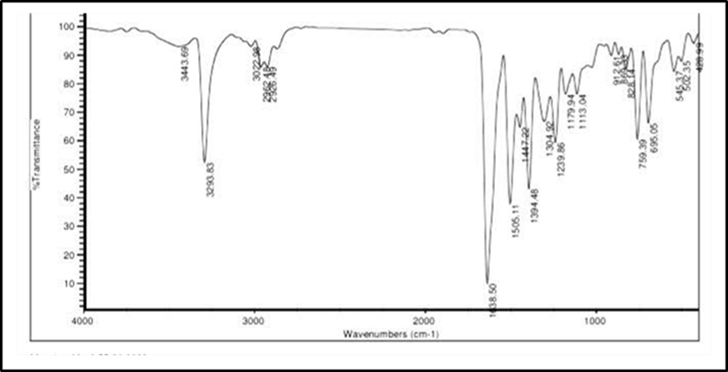


Figure S22: FT-IR spectrum of compound 2k


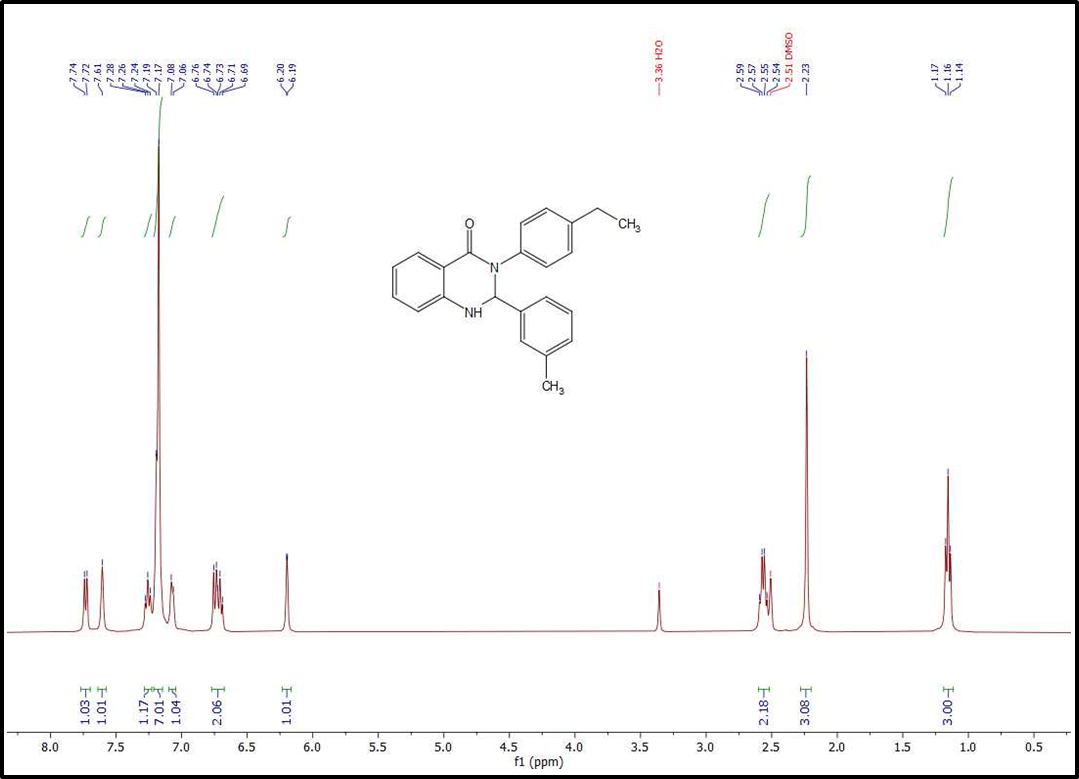


Figure S23: ^1^H NMR spectrum of compound 2k


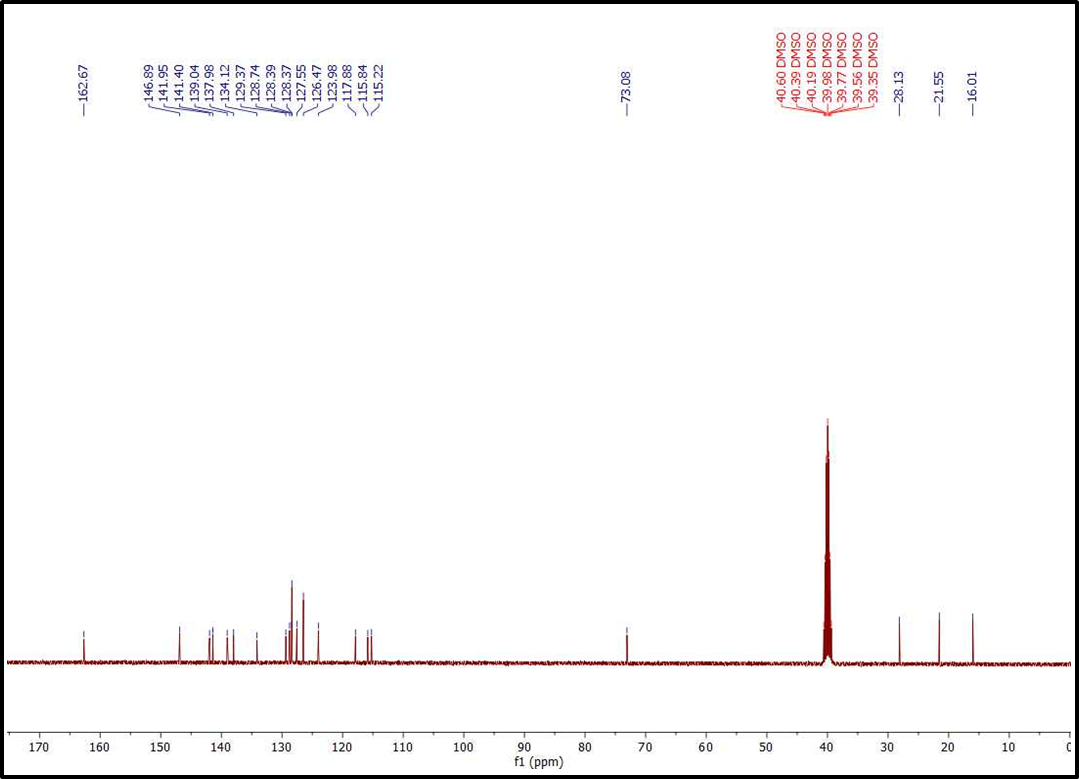


Figure S24: ^13^C NMR spectrum of compound 2k

**3-(5-chloro-2-hydroxyphenyl)-2-(m-tolyl)-2,3-dihydroquinazolin-4(1H)-one** (2l)

Brown solid; IR (KBr) ν (cm^−1^): 3344 (N–H), 3211 (O–H), 1614 (N–C = O). ^1^H NMR (400 MHz, DMSO-d6) δ 10.06 (s, 1H, OH), 7.71 (d, J = 7.8 Hz, 1H), 7.46 – 7.23 (m, 7H), 7.05 (dd, J = 8.7, 2.6 Hz, 1H), 6.93 (d, J = 2.7 Hz, 1H), 6.86 – 6.70 (m, 3H), 6.18 (s, 1H); ^13^C NMR (100 MHz, DMSO-d6) δ 162.85, 152.83, 147.93, 140.09, 134.16, 130.70, 129.21, 128.67, 128.53, 128.40, 127.77, 121.74, 117.94, 117.81, 115.03, 114.90, 73.02 ppm; Anal. calcd. For C_21_H_17_ClN_2_O_2_: C, 69.14; H, 4.70; and N, 7.68, Found: C, 69.18; H, 4.72; and N, 7.68


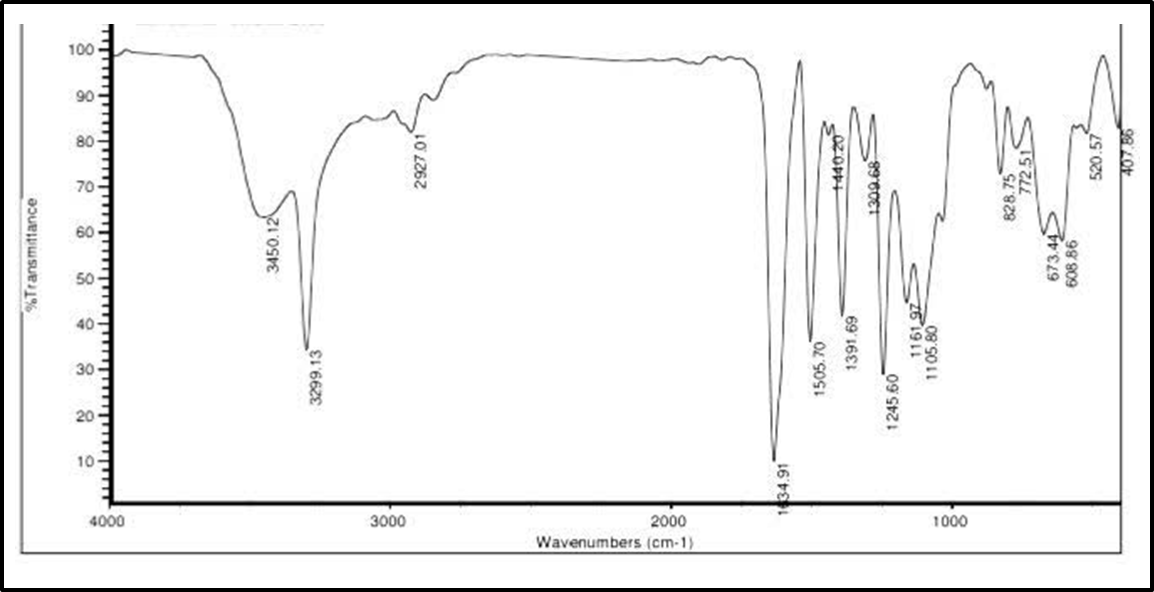


Figure S25: FT-IR spectrum of compound 2l


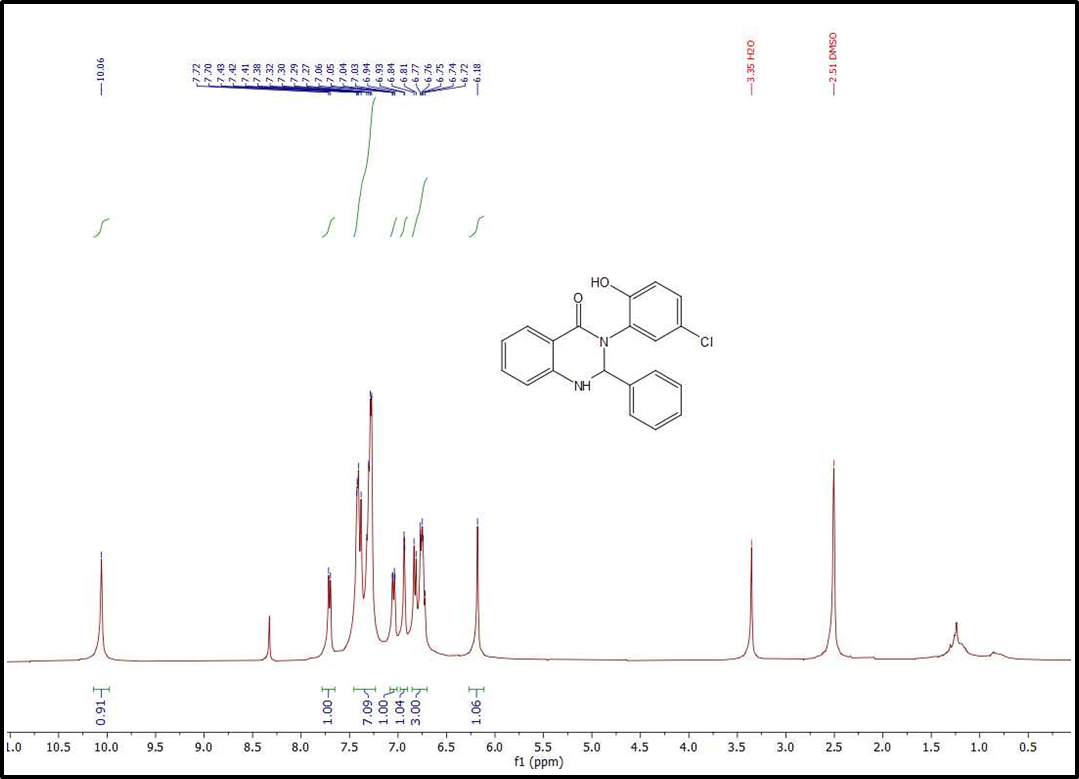


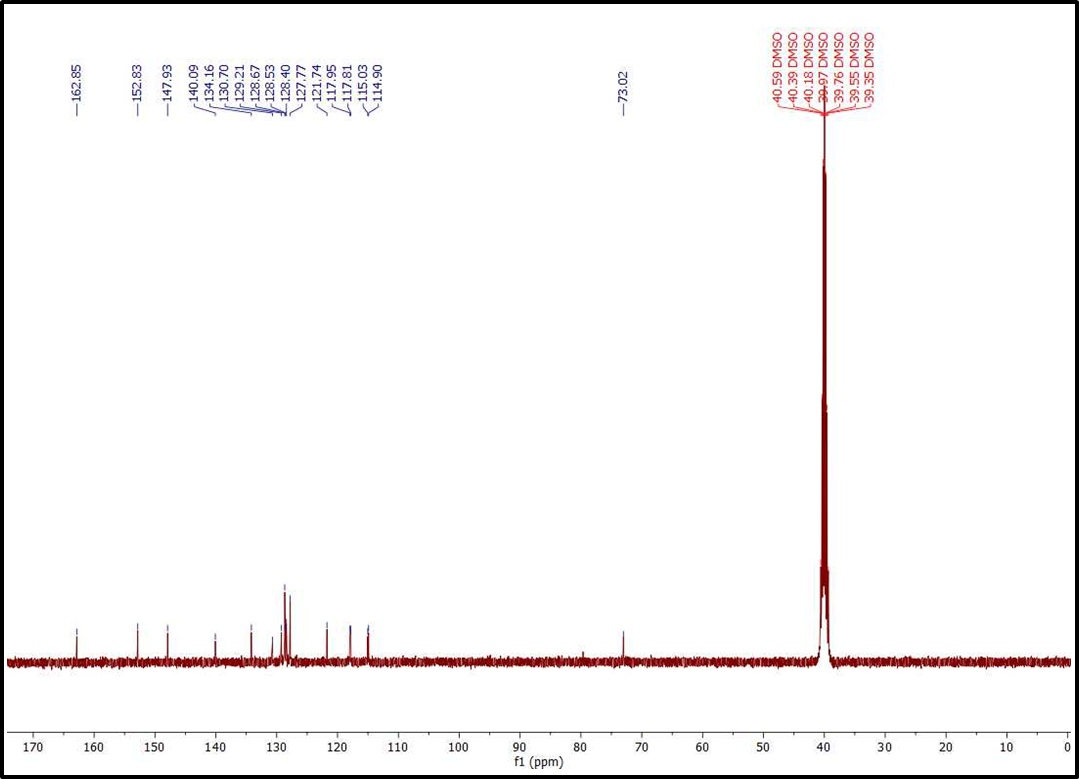


Figure S26: ^1^H NMR spectrum of compound 2l

Figure S27: ^13^C NMR spectrum of compound 2l

**2-(5-(4-chlorophenyl)furan-2-yl)-3-(p-tolyl)-2,3-dihydroquinazolin-4(1H)-one** (3a)


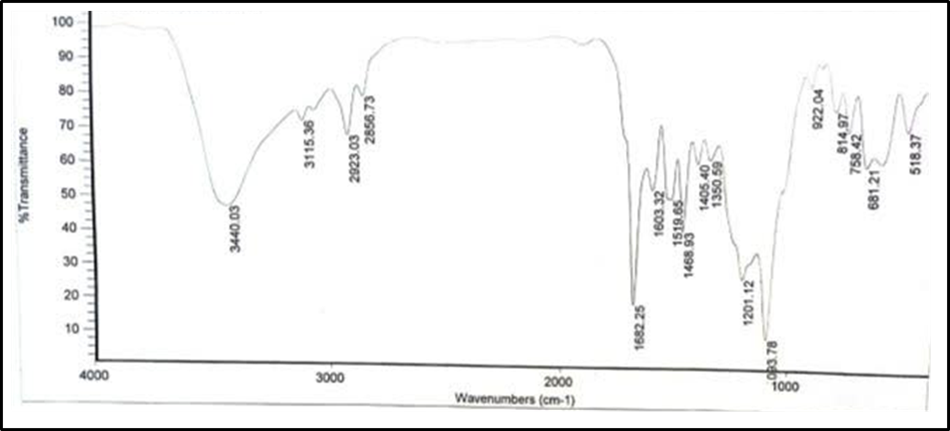
Off-white solid; IR (KBr) ν (cm^−1^): 3440 (N–H), 1682 (N–C = O). ^1^H NMR (400 MHz, DMSO-d6) δ 7.75 (dd, J = 7.6, 2.1 Hz, 1H), 7.69 (d, J = 3.1 Hz, 1H, NH), 7.54 (d, J = 8.6 Hz, 2H), 7.46 (d, J = 8.5 Hz, 2H), 7.32 (t, J = 8.4 Hz, 1H), 7.26 (d, J = 8.0 Hz, 2H), 7.21 (d, J = 8.1 Hz, 2H), 6.86 (dd, J = 5.9, 2.4 Hz, 2H), 6.78 (t, J = 7.5 Hz, 1H), 6.38 (d, J = 3.4 Hz, 1H), 6.27 (d, J = 3.0 Hz, 1H), 2.31 (s, 3H); ^13^C NMR (100 MHz, DMSO-d6) δ 162.30, 153.62, 152.04, 147.05, 138.49, 136.22, 134.07, 132.49, 129.70, 129.41, 129.22, 128.30, 126.79, 125.42, 118.42, 116.22, 115.42, 110.99, 107.55, 67.01, 21.04 ppm; MS (*m/z*): 415 (*M*+); Anal. calcd. For C_25_H_19_ClN_2_O_2_: C, 72.37; H, 4.62; and N, 6.75, Found: C, 72.37; H, 4.65; and N, 6.70

Figure S28: FT-IR spectrum of compound 3a


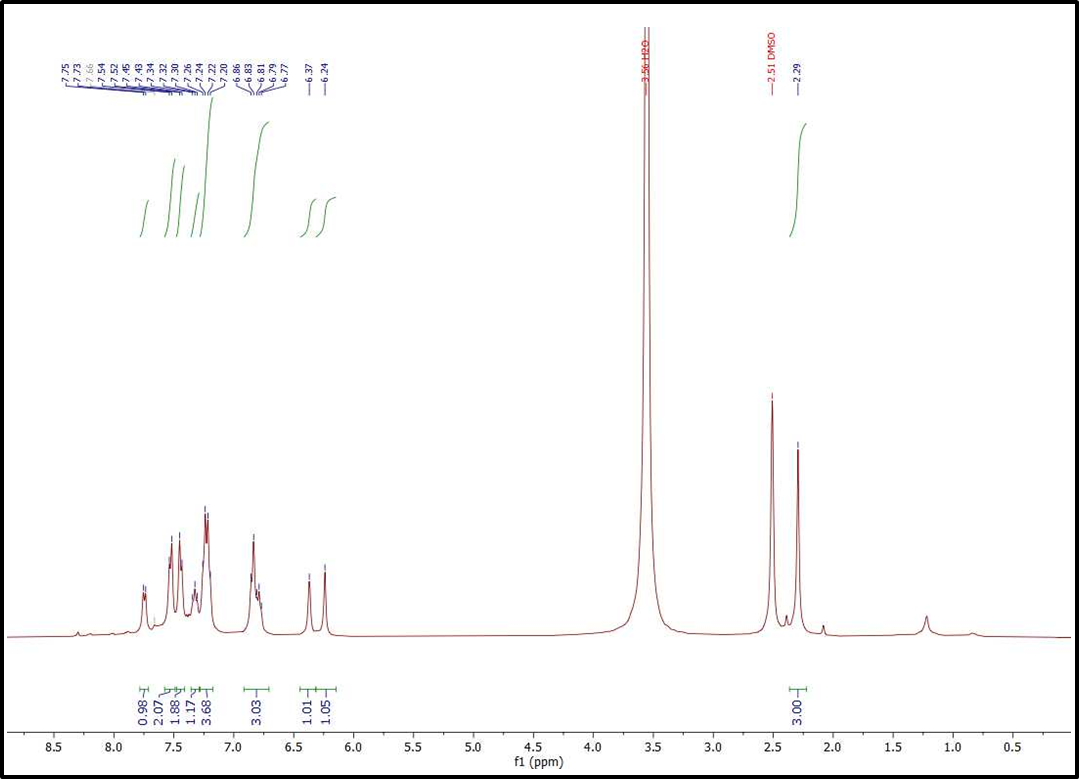

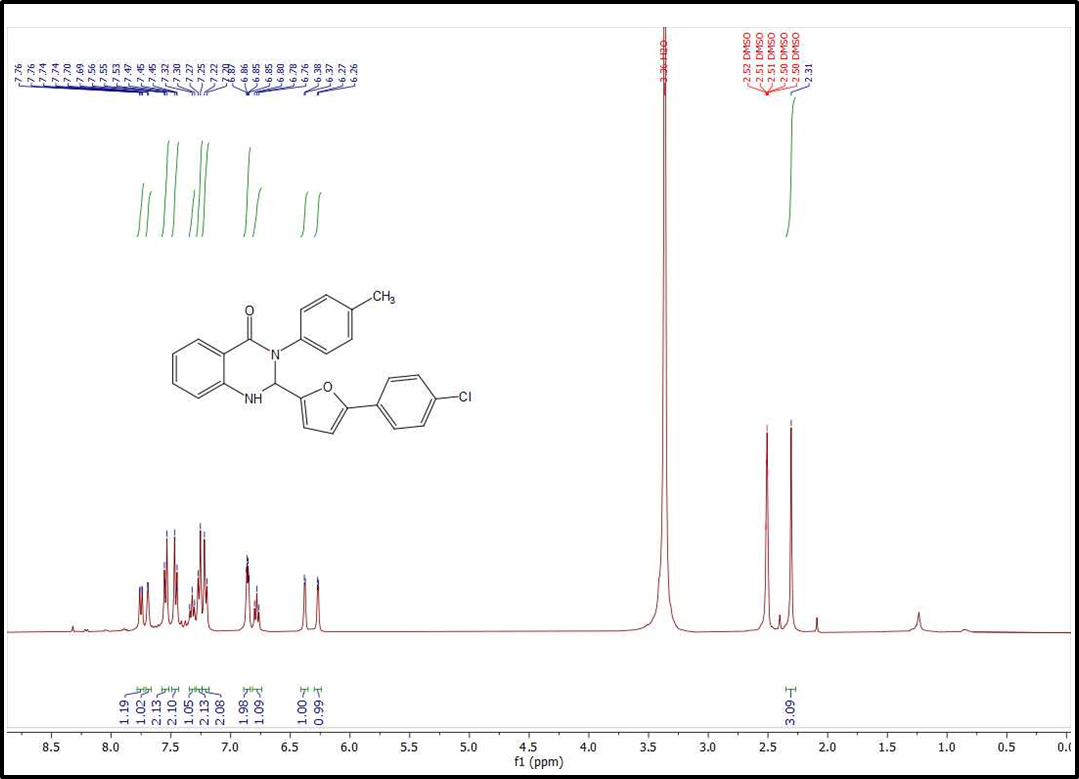


Figure S29: ^1^H NMR spectrum of compound 3a

Figure S30: ^1^H NMR D_2_O spectrum of compound 3a

**
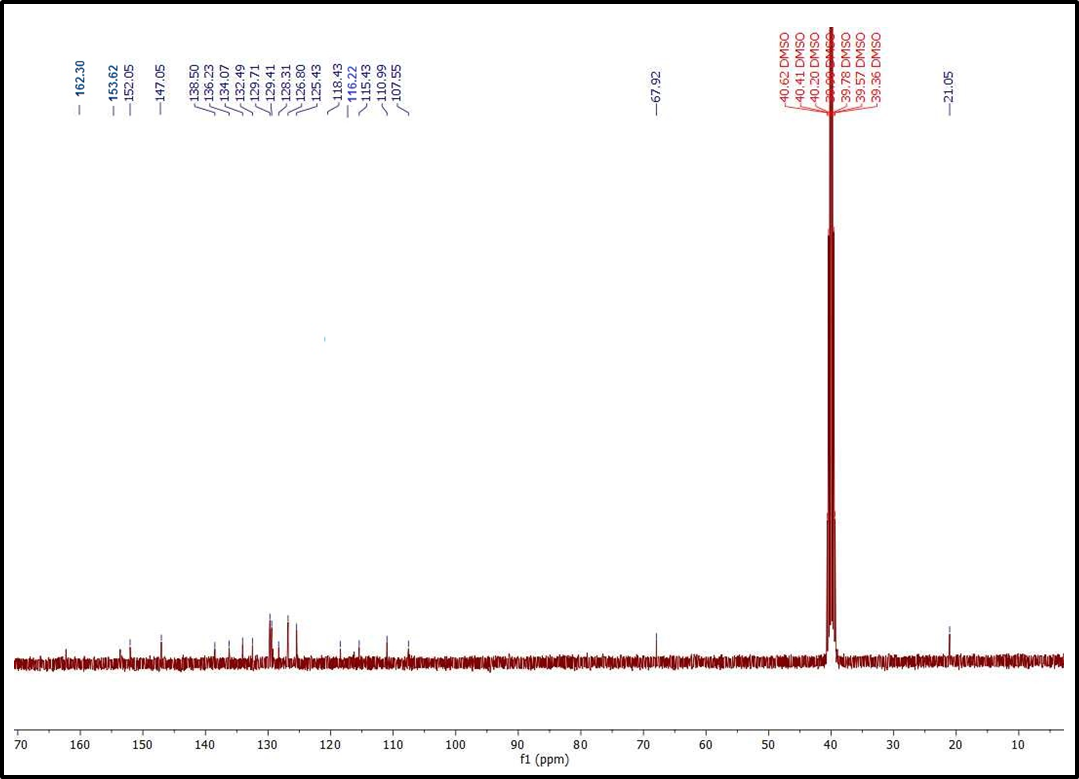
**

Figure S31: ^13^C NMR spectrum of compound 3a

**2-(5-(4-bromophenyl)furan-2-yl)-3-(p-tolyl)-2,3-dihydroquinazolin-4(1H)-one** (3b)

Off*-*white solid; IR (KBr) ν (cm^−1^): 3315 (N–H), 1632 (N–C = O). ^1^H NMR (400 MHz, DMSO-d6) δ 7.75 (d, *J* = 7.8 Hz, 1H), 7.69 (d, *J* = 3.1 Hz, 1H, NH), 7.59 (d, J = 8.4 Hz, 1H), 7.48 (d, *J* = 8.6 Hz, 2H), 7.32 (t, *J* = 7.6 Hz, 1H), 7.26 (d, *J* = 8.0 Hz, 2H), 7.21 (d, *J* = 8.2 Hz, 2H), 6.90 – 6.83 (m, 2H), 6.78 (t, *J* = 7.6 Hz, 1H) 6.38 (d, *J* = 3.4 Hz, 1H), 6.27 (d, *J* = 2.9 Hz, 1H), 2.31 (s, 3H); ^13^C NMR (100 MHz, DMSO-d6) δ 162.32, 153.64, 152.09, 147.03, 138.48, 136.25, 134.09, 132.29, 129.72, 129.54, 128.31, 126.80, 125.69, 121.05, 118.46, 116.22, 115.43, 111.02, 107.62, 67.93, 21.04 ppm; MS (*m/z*): 460 (*M* +); Anal. calcd. For C_25_H_19_BrN_2_O_2_: C, 65.37; H, 4.17; and N, 6.10, Found: C, 65.37; H, 4.17; and N, 6.12


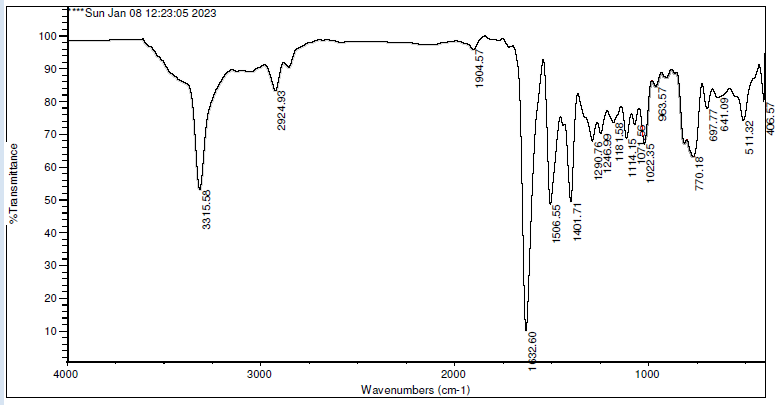


Figure S32: FT-IR spectrum of compound 3b


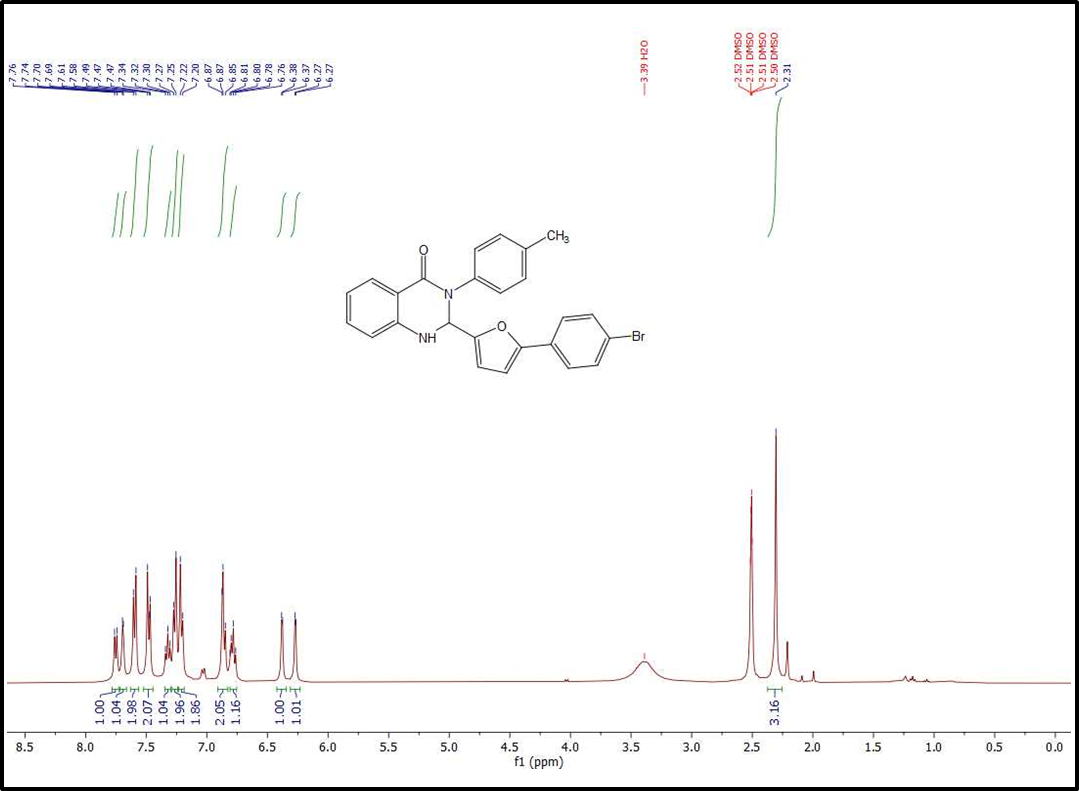


Figure S33: ^1^H NMR spectrum of compound 3b


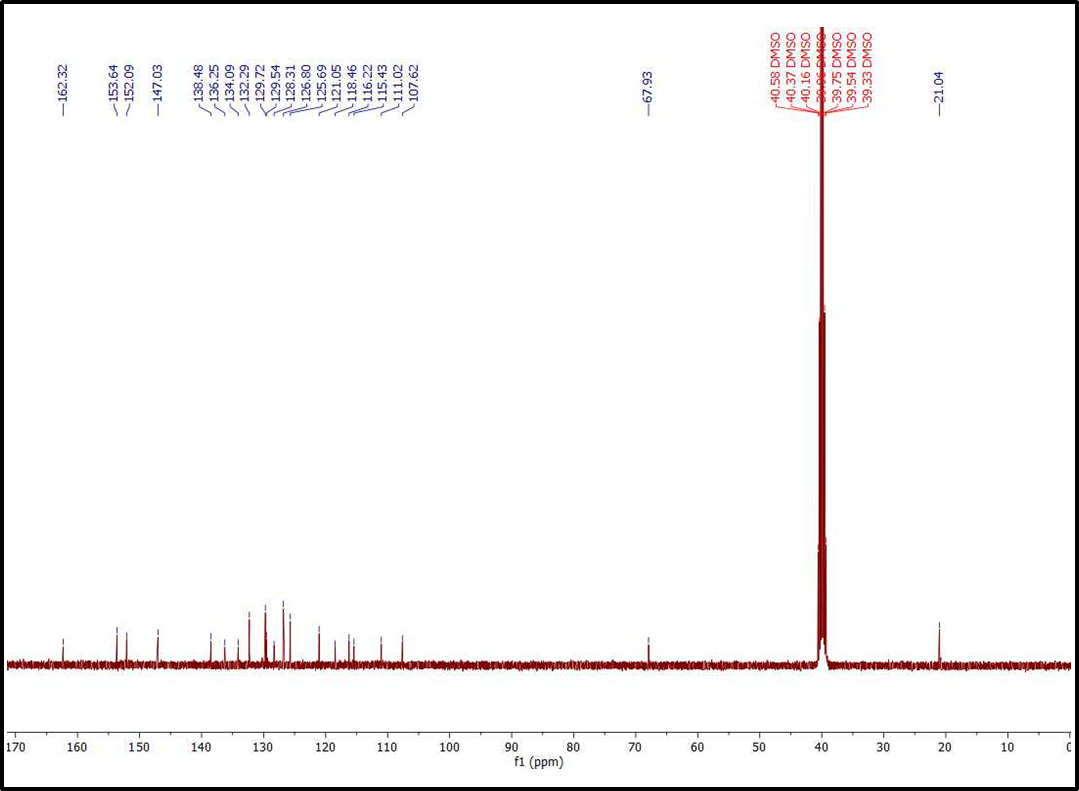


Figure S34: ^13^C NMR spectrum of compound 3b

**2-(5-(2,4-dichlorophenyl)furan-2-yl)-3-(p-tolyl)-2,3-dihydroquinazolin-4(1H)-one** (3c)

Off-white solid; IR (KBr) ν (cm^−1^): 3277 (N–H), 1682 (N–C = O). 1H NMR (400 MHz, CDCl_3_) δ 8.05 (d, J = 7.9 Hz, 1H), 7.48 (d, J = 8.5 Hz, 1H), 7.43 (d, J = 2.2 Hz, 1H, NH), 7.38 – 7.31 (m, 2H), 7.27 (d, J = 8.0 Hz, 2H), 7.20 (d, J = 8.3 Hz, 3H), 6.99 – 6.90 (m, 2H), 6.74 (d, J = 8.1 Hz, 1H), 6.42 (d, J = 3.5 Hz, 1H), 6.06 (s, 1H), 2.36 (s, 3H); ^13^C NMR (100 MHz, CDCl_3_) δ 162.65, 152.30, 149.42, 148.57, 145.26, 138.03, 136.95, 135.75, 133.36, 130.56, 130.42, 129.85, 129.76, 128.89, 128.43, 127.27, 126.31, 119.91, 117.29, 115.19, 111.74, 110.83, 68.65, 21.09 ppm; MS (m/z): 450 (M +); Anal. calcd. For C_25_H_18_Cl_2_N_2_O_2_: C, 66.83; H, 4.04; and N, 6.23, Found: C, 66.83; H, 4.18; and N, 6.19


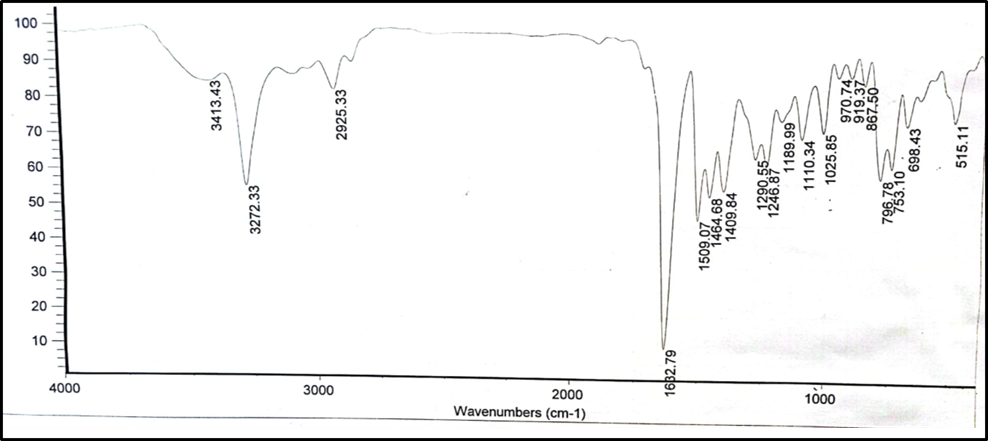


Figure S35: FT-IR spectrum of compound 3c


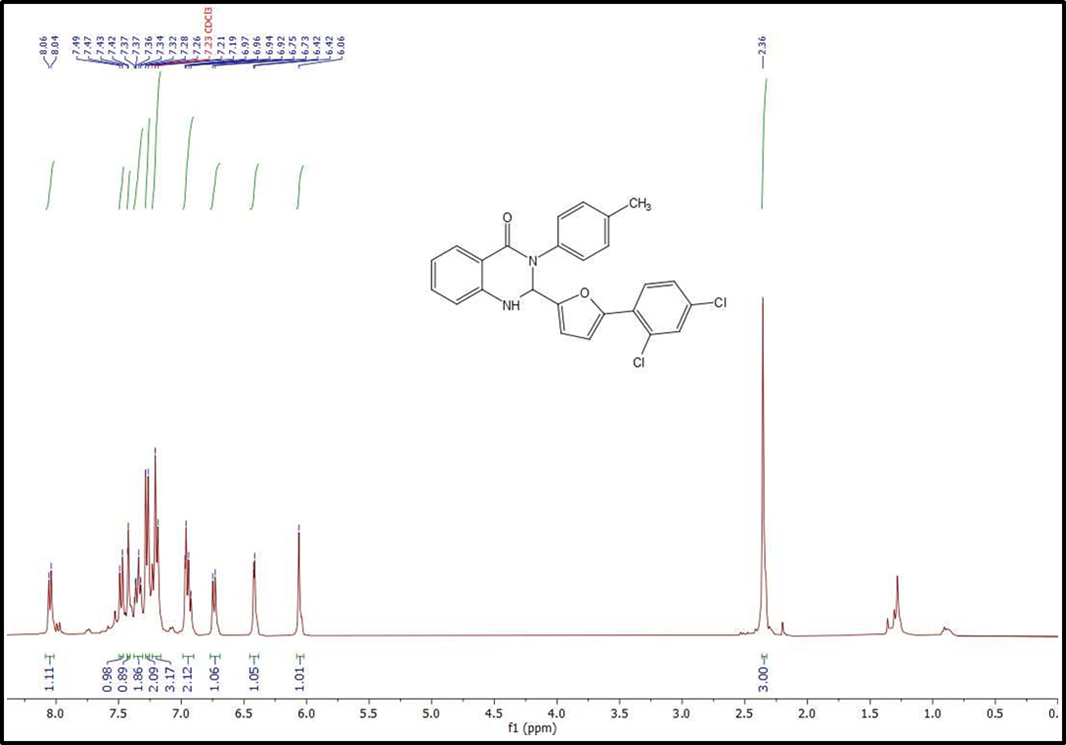


Figure S36: ^1^H NMR spectrum of compound 3c


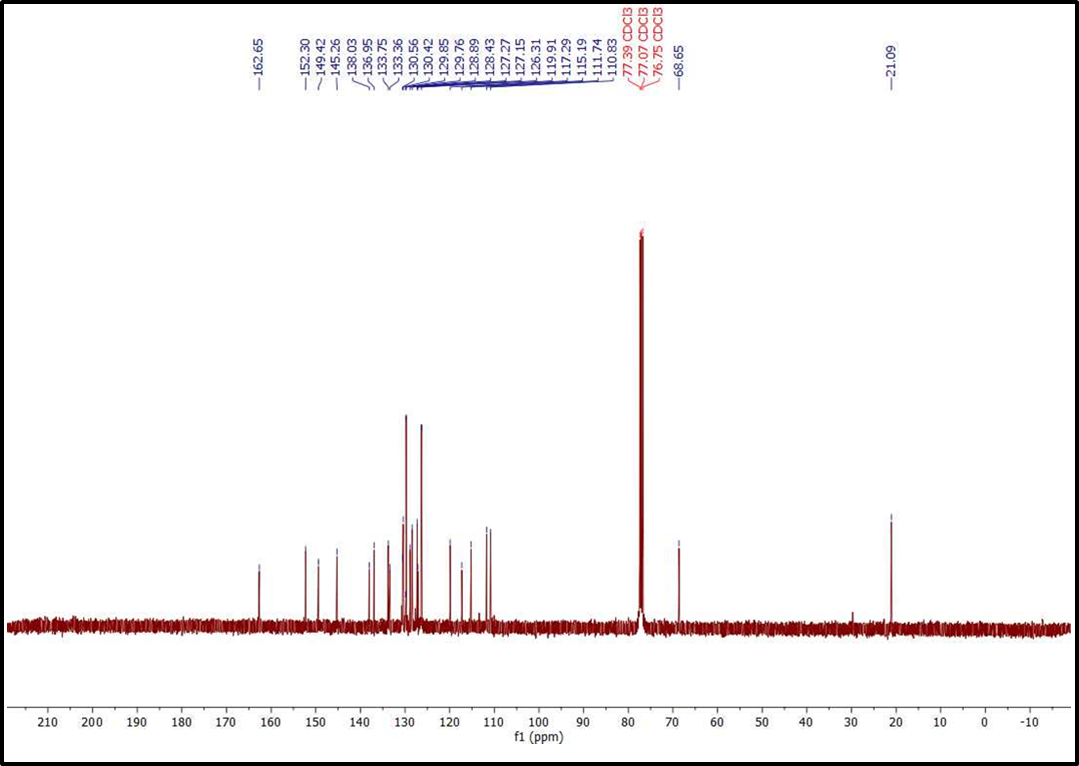


Figure S37: ^13^C NMR spectrum of compound 3c

**2-(5-(2-nitrophenyl)furan-2-yl)-3-(p-tolyl)-2,3-dihydroquinazolin-4(1H)-one** (3d)

Off-white solid; IR (KBr) ν (cm^−1^): 3425 (N–H), 1681 (N–C = O). ^1^H NMR (400 MHz, DMSO-d6) δ 7.99-7.43 (m, 6H), 7.41-7.00 (m, 5H), 6.96 – 6.55 (m, 3H), 6.40 (s, 1H), 6.23 (s, 1H), 2.31 (s, 3H); ^13^C NMR (100 MHz, DMSO-d6) δ 162.07, 155.10, 148.07, 146.83, 138.43, 136.24, 135.55, 134.09, 130.82, 129.72, 129.35, 126.77, 124.94, 124.52, 122.95, 121.04, 118.45, 116.08, 115.39, 111.06, 110.93, 67.76, 21.05 ppm; MS (m/z): 426 (M +); Anal. calcd. For C_25_H_19_N_3_O_4_: C,70.58; H, 4.50; and N, 9.88, Found: C, 70.58; H, 4.69; and N, 9.78


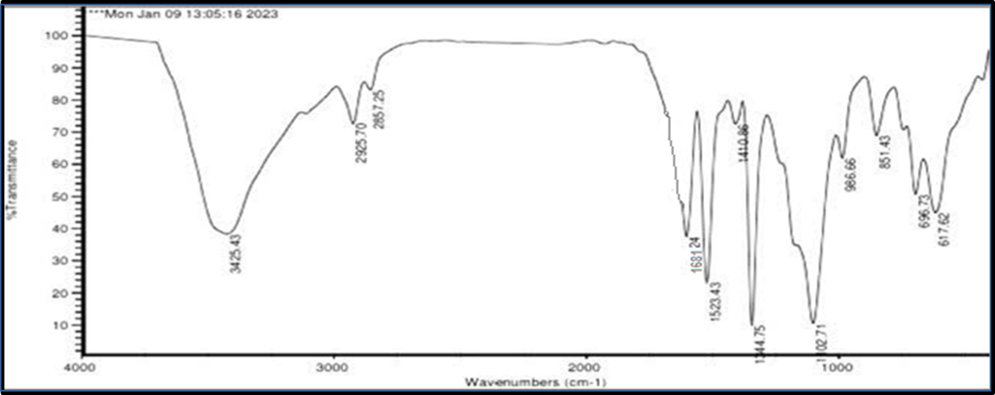


Figure S38: FT-IR spectrum of compound 3d


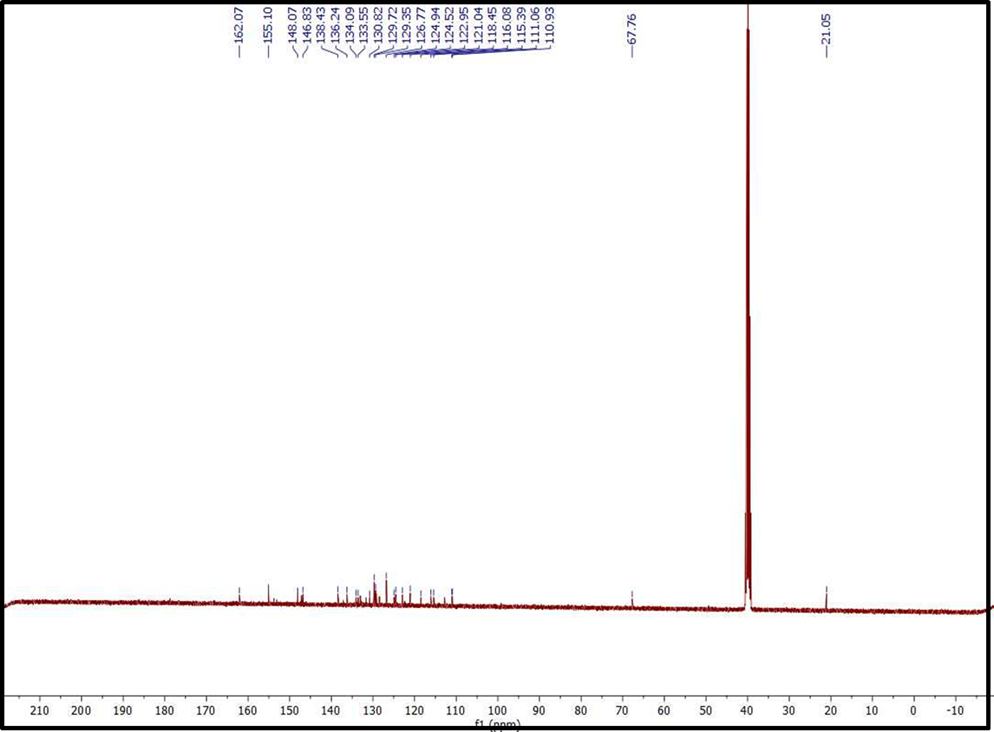

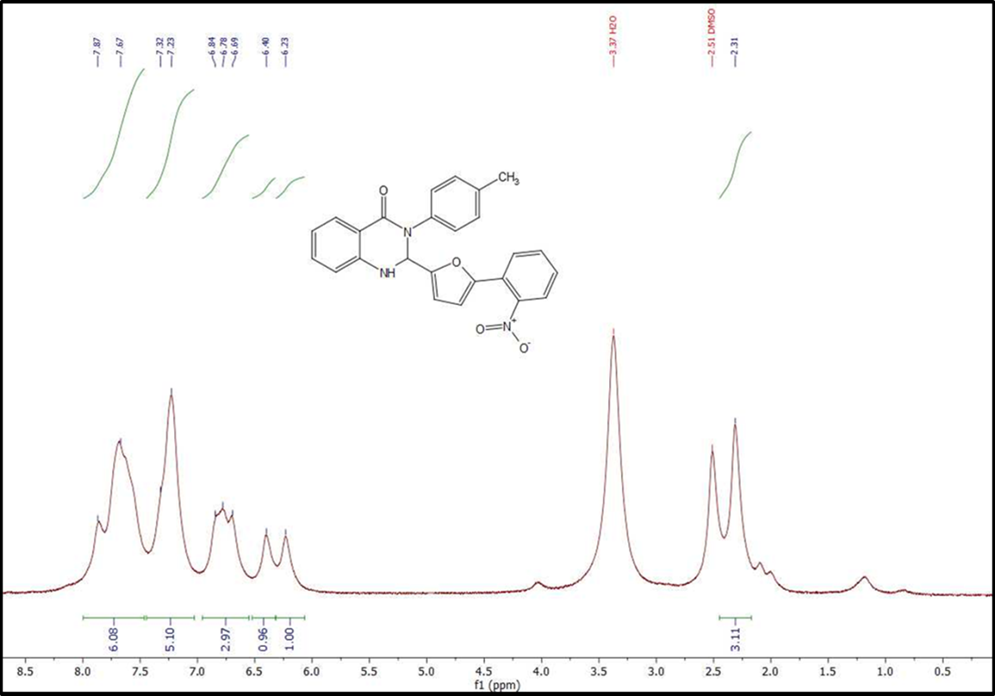


Figure S39: ^1^H NMR spectrum of compound 3d

Figure S40: ^13^C NMR spectrum of compound 3d

**2-(5-(2,4-dichlorophenyl)furan-2-yl)-3-(4-ethylphenyl)-2,3-dihydroquinazolin-4(1H)-one** (3e)


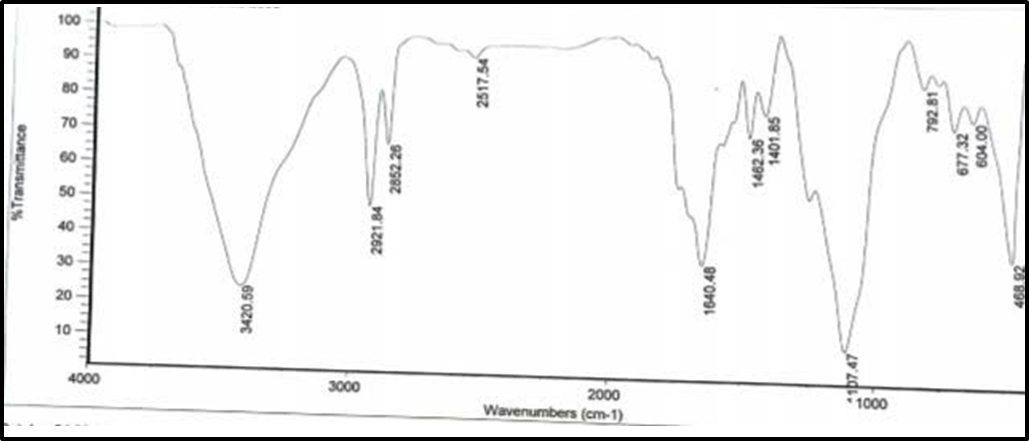
Off-white solid; IR (KBr) ν (cm^−1^): 3420 (N–H), 1640 (N–C = O). 1H NMR (400 MHz, CDCl_3_) δ 8.06 (dd, J = 7.9, 1.5 Hz, 1H), 7.49 (d, J = 8.5 Hz, 1H), 7.43 (d, J = 2.1 Hz, 1H, NH), 7.38 – 7.28 (m, 5H), 7.25 – 7.22 (m, 2H), 7.01 – 6.92 (m, 2H), 6.75 (d, J = 8.0 Hz, 1H), 6.44 (d, J = 3.5 Hz, 1H), 6.09 (s, 1H), 2.66 (q, J = 7.6 Hz, 2H), 1.25 (t, J = 7.6 Hz, 3H); 13C NMR (100 MHz, CDCl_3_)δ 162.51, 152.25, 149.51, 145.02, 143.22, 138.16, 133.74, 130.65, 130.46, 129.00, 128.60, 128.45, 127.28, 127.16, 126.29, 120.13, 117.21, 115.14, 111.76, 110.91, 68.70, 28.68, 15.48 ppm; MS (m/z): 464 (M +); Anal. calcd. For C_26_H_20_Cl_2_N_2_O_2_: C, 67.39; H, 4.35; and N, 6.05, Found: C, 67.42; H, 4.35; and N, 6.05

Figure S41: FT-IR spectrum of compound 3e


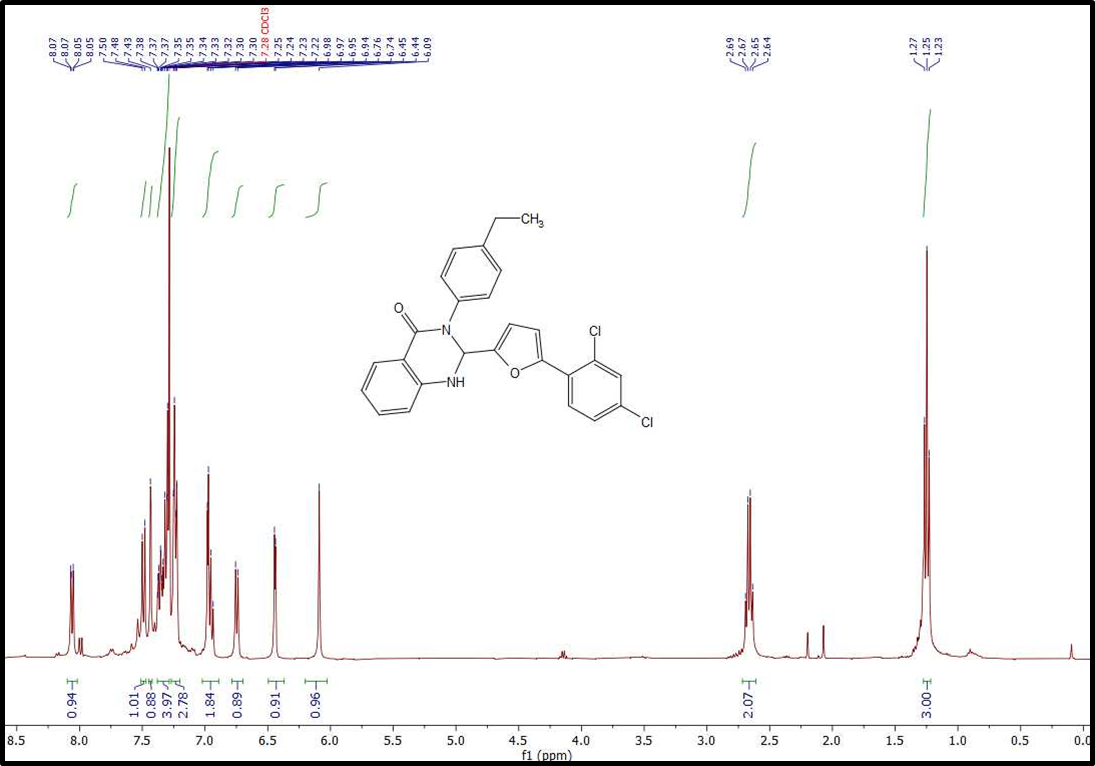


Figure S42: ^1^H NMR spectrum of compound 3e


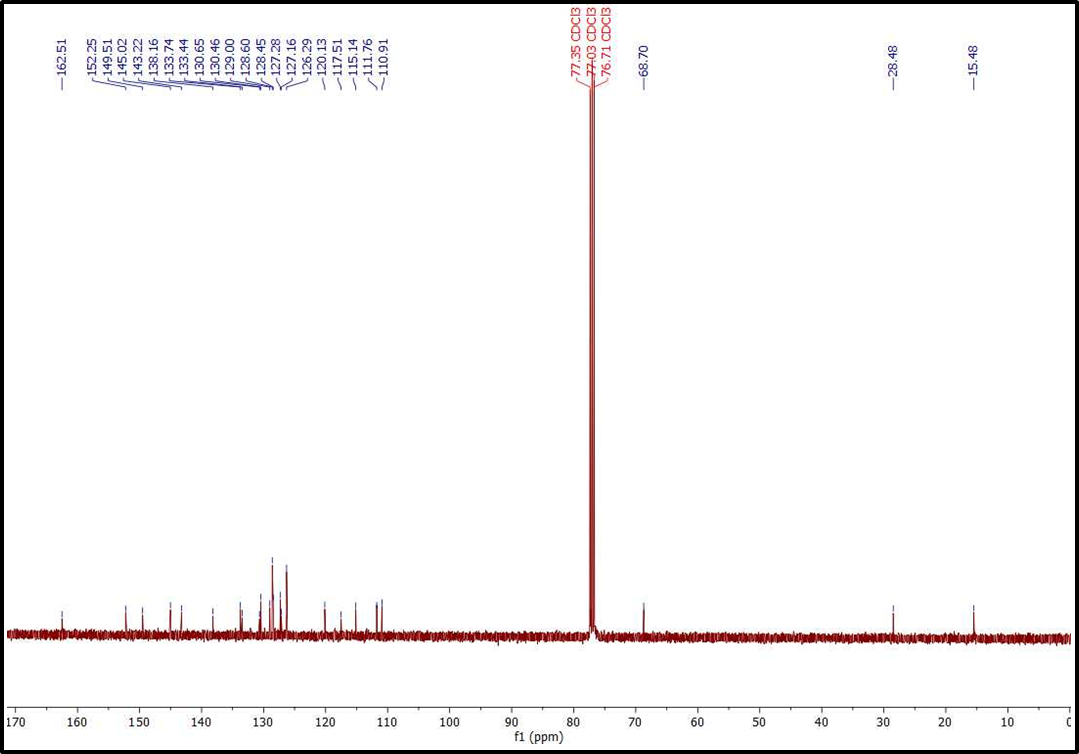


Figure S43: ^13^C NMR spectrum of compound 3e

**2-(5-(2,4-dichlorophenyl)furan-2-yl)-3-phenyl-2,3-dihydroquinazolin-4(1H)-one** (3f)

Off*-*white solid; IR (KBr) ν (cm^−1^): 3277 (N–H), 1684 (N–C = O). ^1^H NMR (400 MHz, DMSO-d6) δ 7.82 – 7.73 (m, 2H), 7.68 (s, 1H, NH), 7.60 (d, *J* = 8.8 Hz, 1H), 6.79 (t, *J* = 7.4 Hz, 1H), 6.47 (s, 1H), 6.37 (s, 1H); ^13^C NMR (100 MHz, DMSO-d6) δ 162.29, 153.90, 148.57, 147.07, 140.99, 140.07, 134.19, 133.03, 130.62, 130.17, 129.27, 129.11, 128.39, 128.20, 127.42, 126.83, 118.53, 116.19, 115.51, 112.49, 110.98, 67.76 ppm; MS (m/z): 436 (M +); Anal. calcd. For C_24_H_16_Cl_2_N_2_O_2_: C, 66.22; H, 3.70; and N, 6.44, Found: C, 66.22; H, 3.78; and N, 6.42


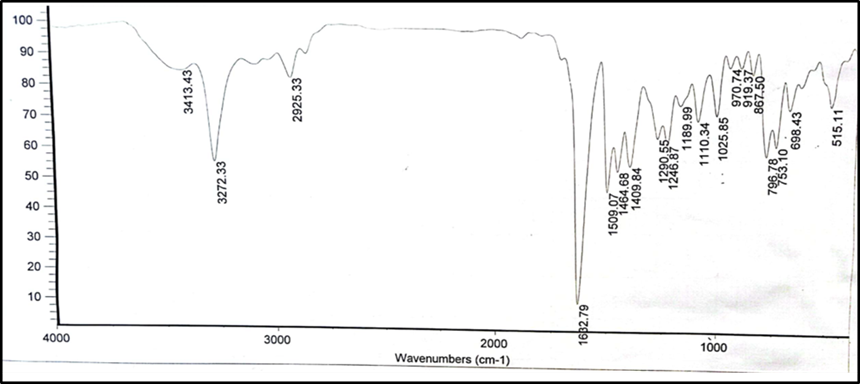


Figure S44: FT-IR spectrum of compound 3f


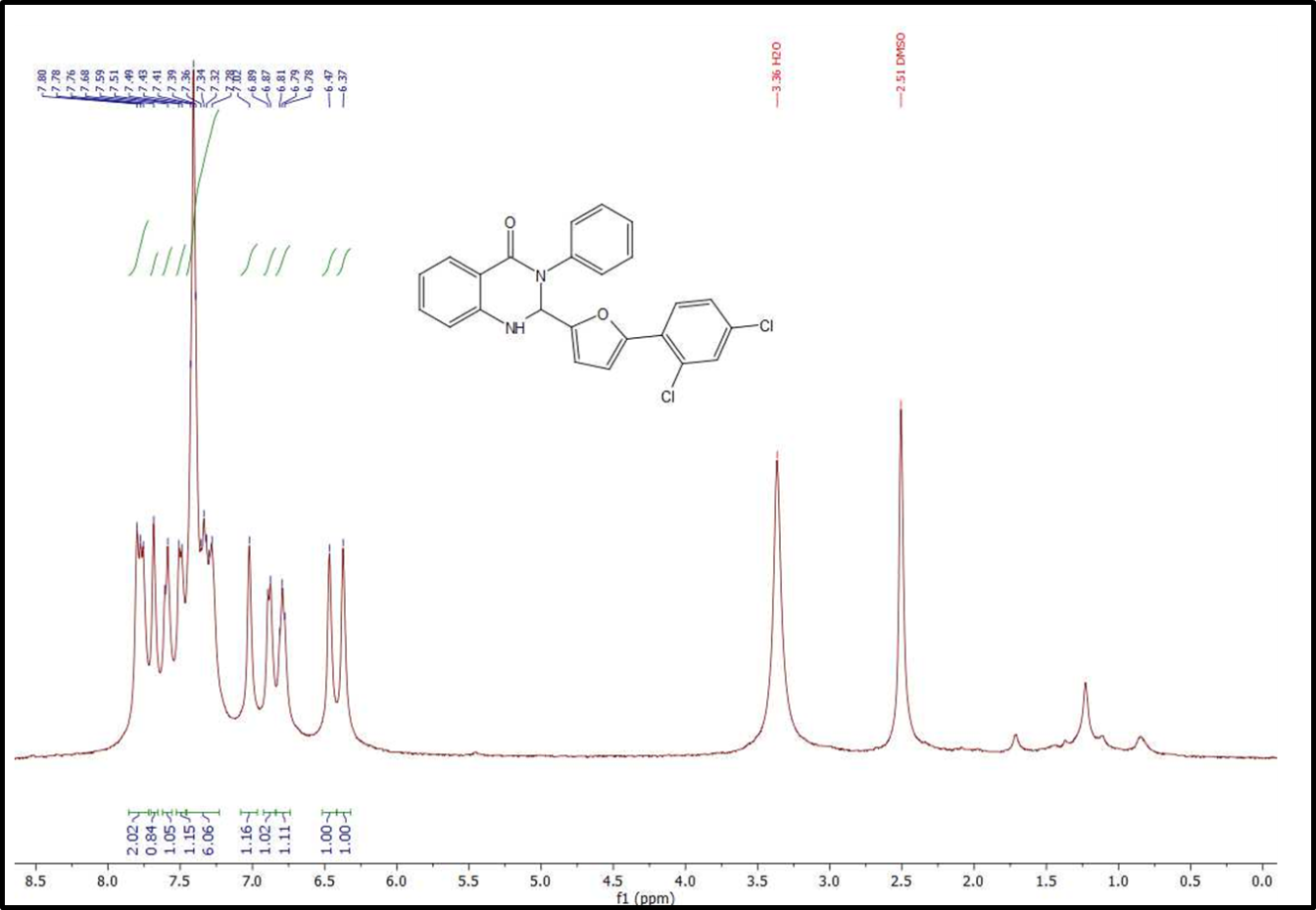


Figure S45: ^1^H NMR spectrum of compound 3f


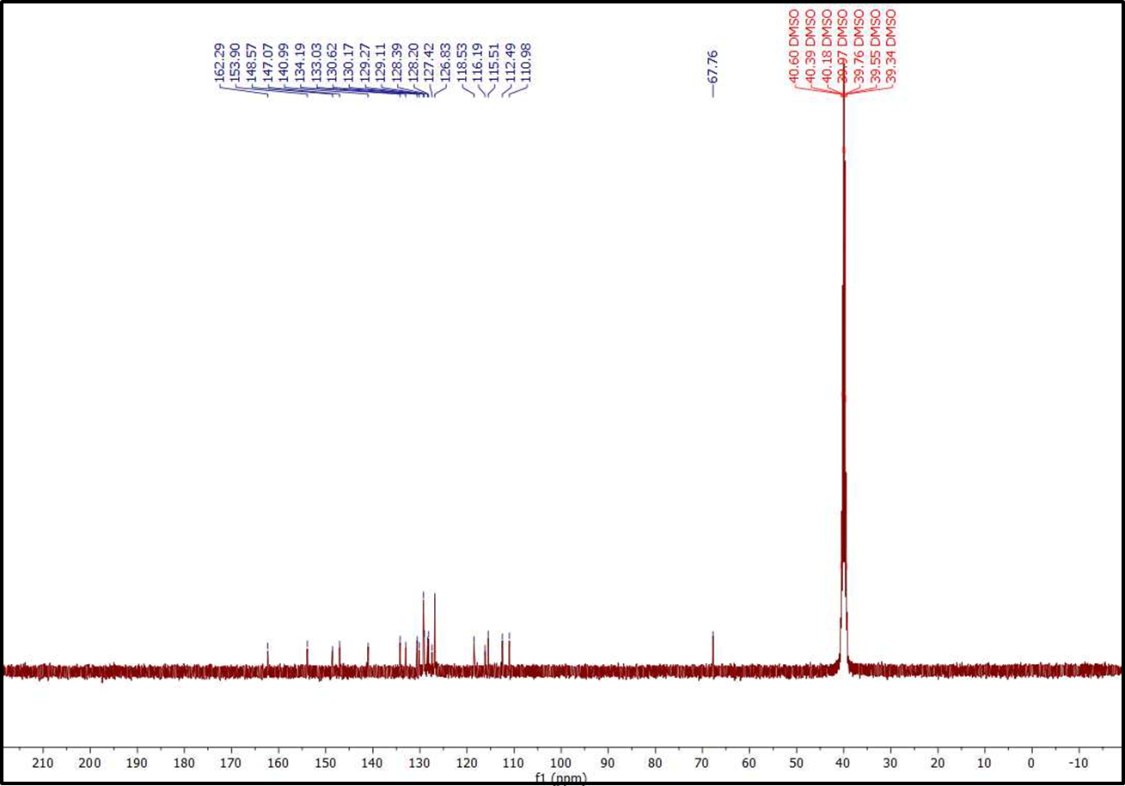


Figure S46: ^13^C NMR spectrum of compound 3f
